# Stretchable, Ultratough, and Intrinsically Self‐Extinguishing Elastomers with Desirable Recyclability

**DOI:** 10.1002/advs.202207268

**Published:** 2023-01-22

**Authors:** Yijiao Xue, Jinyou Lin, Tao Wan, Yanlong Luo, Zhewen Ma, Yonghong Zhou, Bryan T. Tuten, Meng Zhang, Xinyong Tao, Pingan Song

**Affiliations:** ^1^ Institute of Chemical Industry of Forest Products Chinese Academy of Forestry (CAF) Nanjing 210042 China; ^2^ Shanghai Advanced Research Institute Chinese Academy of Sciences Shanghai 201204 China; ^3^ School of Materials Science and Engineering The University of New South Wales Sydney NSW 2502 Australia; ^4^ College of Science Nanjing Forestry University Nanjing 210037 China; ^5^ Department of Polymer Materials School of Materials Science and Engineering Tongji University Shanghai 201804 China; ^6^ Centre for Materials Science School of Chemistry and Physics Queensland University of Technology Brisbane QLD 4000 Australia; ^7^ College of Materials Science and Engineering Zhejiang University of Technology Hangzhou 310014 China; ^8^ Centre for Future Materials Unviersity of Southern Queensland Springfield 4300 Australia; ^9^ School of Agriculture and Environmental Science Unviersity of Southern Queensland Springfield 4300 Australia

**Keywords:** mechanical robustness, polyurethane elastomers, self‐extinguishing, strain‐hardening, stretchable device, *π*–*π* stacking

## Abstract

Advanced elastomers are increasingly used in emerging areas, for example, flexible electronics and devices, and these real‐world applications often require elastomers to be stretchable, tough and fire safe. However, to date there are few successes in achieving such a performance portfolio due to their different governing mechanisms. Herein, a stretchable, supertough, and self‐extinguishing polyurethane elastomers by introducing dynamic *π*–*π* stacking motifs and phosphorus‐containing moieties are reported. The resultant elastomer shows a large break strain of ≈2260% and a record‐high toughness (ca. 460 MJ m^−3^), which arises from its dynamic microphase‐separated microstructure resulting in increased entropic elasticity, and strain‐hardening at large strains. The elastomer also exhibits a self‐extinguishing ability thanks to the presence of both phosphorus‐containing units and *π*–*π* stacking interactions. Its promising applications as a reliable yet recyclable substrate for strain sensors are demonstrated. The work will help to expedite next‐generation sustainable advanced elastomers for flexible electronics and devices applications.

## Graphical abstract

By introducing well‐designed dynamic *π*–*π* stacking motifs and phosphorus‐containing moieties, a mechanically strong, supertough and fire retardant polyurethane elastomer is developed, demonstrating a high tensile strength of ≈57 MPa, a large break strain of ≈2260%, a record‐high toughness (ca. 460 [±15] MJ m^−3^) and a self‐extinguishing ability, which hold great promise for flexible electronics and devices applications.

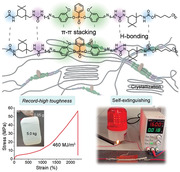

## 1 Introduction

Due to their high entropy elasticity, thermoplastic polyurethane (PU) elastomers can dissipate mechanical energy and restore their original shape and mechanical properties after undergoing repeated shape deformations. These features of PU elastomers have garnered interests in many emerging fields such as aircraft tires, flexible electronics, flexible electronics, and stretchable devices.^[^ ^1^, ^2^, ^3^, ^4^ ^]^ With intense effort, great advances have been made in the design of strong, tough, and even healable PU elastomers.^[^ ^5^, ^6^, ^7^ ^]^ However, good fire retardancy is often equally important to ensure their reliable applications, for example, flexible electronics and devices. Hence, it is highly desirable to create stretchable, tough, and fire‐safe PU elastomers to enable their real‐world applications.

To achieve fire retardancy, the most facile strategy is the inclusion of flame retardants into PU elastomers through melting or solution blending. This approach, however, normally leads to reduced mechanical properties (strength and break strain) of the final elastomers, owing to the poor interfacial incompatibility between fire retardants and PU chains.^[^ ^8^, ^9^, ^10^ ^]^ In comparison, chemically bonding fire retardant groups can lead to intrinsically fire retardant PU elastomers that often show robust mechanical properties. For example, Kang et al. reported sulfur‐based segmented multi‐block polyurethanes (SPUs) that exhibit a desired V‐0 rating in UL‐94 testing, in addition to acceptable tensile strength (13–24 MPa) and ductility (348% strain at break).^[^ ^11^ ^]^ Xia et al. synthesized a boron‐containing chain extender of diglyceride borate for improving flame retardancy of PU. The final elastomer shows a high limiting oxygen index (LOI) of 30.0%, but relatively low tensile strength (3.06 MPa) and strain at break (178%).^[^ ^12^ ^]^ Despite good fire retardancy, the mechanical properties of these inherently fire‐safe PU elastomers remain incomparable to those of most high‐performance non‐fire retardant counterparts.^[^ ^5^, ^6^, ^7^ ^]^ Therefore, to date it remains a grand challenge to achieve the combination of both high stretchability, high strength, great toughness, and excellent fire retardancy into one PU elastomer system.^[^ ^5^, ^13^, ^14^, ^15^, ^16^ ^]^

To address the challenge, P(O)R_1_(OR_2_)_2_ groups with a low molar contribution to the heat release capacity is first rationally designed for imparting the fire retardant function to PU elastomers.^[^ ^16^, ^17^, ^18^ ^]^ Meanwhile, in order to enable a large deformation ability in the benzene‐rich structure of the elastomer, dynamic covalent bonds (C=N), Schiff base, is then introduced to yield *π*–*π* stacking interactions which can become stronger if combined with benzene rings.^[^ ^13^, ^19^, ^20^ ^]^ The dynamic *π*–*π* interactions are expected to induce the formation of a dynamic microphase‐separation structure that can serve as the crosslinking sites for the target PU elastomers, thus giving rise to great toughness and high strength.

Through this design, the target PU elastomer exhibits a record‐high toughness of ≈460.4 MJ m^−3^, a strain at break as high as ≈2300% and a tensile strength of ≈57 MPa, arising from its large entropic elasticity of polymer chains at small strains and the strain‐induced crystallization of soft segments at large strains. Moreover, the elastomer achieves a high LOI value of 32.5% and a desired UL‐94 V‐0 rating, exhibiting a self‐extinguishing behavior, because of the synergy between the P‐containing groups and Schiff base (C=N). Such a performance portfolio is unprecedentedly superior to that of previous PU elastomers. The as‐designed PU elastomer can be used as a substrate for reliable flexible sensors. Moreover, the elastomer can dissolve in ethanol, thus making it easier to process, compared with commercial PU elastomers that can only dissolve in high boiling solvents, such as *N*,*N*‐dimethylformamide. This work will contribute to the design of next‐generation high‐performance elastomers for their real‐world applications in industry settings, such as e‐skin, electronics and devices.

## 2 Results and Discussion

### 2.1 Materials Design and Characterizations

**Figure 1:**
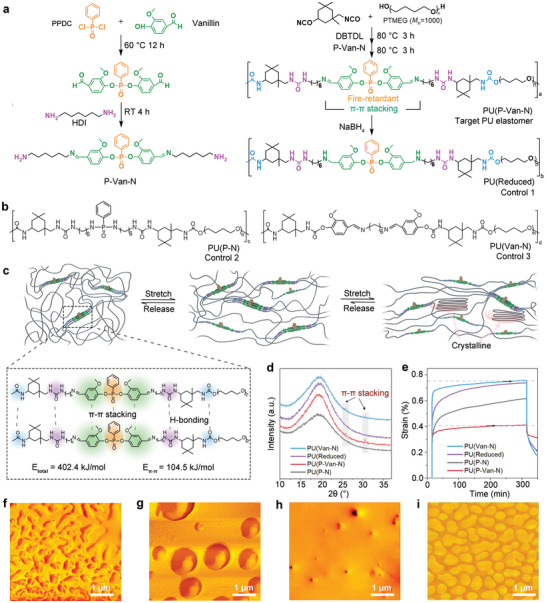
Rational design and characterizations of stretchable, tough and fire retardant polyurethane elastomers, PU(P‐Van‐N). a) Synthesis of the P‐Van‐N, PU(P‐Van‐N), and PU(Reduced) elastomers. b) Structure diagrams for controls of PU(P‐N) and PU(Van‐N). c) Cartoon representation of the proposed mechanism for highly stretchable PU(P‐Van‐N). d) XRD patterns at room temperature. e) Creep behavior at 0.1 MPa and 35 °C for PU(P‐Van‐N), PU(Reduced), PU(P‐N) and PU(Van‐N) and AFM phase images of f) PU(P‐Van‐N), g) PU(Reduced), h) PU(P‐N) and i) PU(Van‐N).

The dilemma of the combining advantageous mechanical properties and fire retardancy in PU elastomers has proven to be a critical challenge for their practical applications.^[^ ^21^ ^]^ To achieve this performance portfolio, an amino‐terminated phosphorus‐containing chain extender (P‐Van‐N) with fire retardant and *π*–*π* stacking groups is first designed to fabricate the target PU elastomer (PU(P‐Van‐N)), for which polytetramethylene glycol (PTMEG, *M*_n_ = 1000 g mol^−1^) is used as the soft segment (see **Figure** **1**a). To investigate the role of the Schiff base (C=N) or *π*–*π* stacking in mechanical and fire retardant properties, PU(P‐Van‐N) is chemically reduced to generate a control elastomer, PU(Reduced). Meanwhile, two other controls, PU(P‐N) and PU(Van‐N) are also synthesized to comparatively examine the roles of both Schiff base and P‐Van‐N moieties (see Figure 1a,b). In addition, PU(OH) and PU(NH_2_) without fire retardant and *π*–*π* stacking groups are also prepared for comparison (Figure S3, Supporting Information).

The presence of characteristic peaks of C=N (1689 cm^−1^), P—O—Ph (913 cm^−1^), and P=O (1272 cm^−1^) bonds suggests the successful synthesis of P‐Van‐N in its infrared (IR) spectrum (Figure S6a, Supporting Information).^[^ ^22^, ^23^ ^]^ Meanwhile, the absorption peak of NCO groups at around 2264 cm^−1^ disappears in the IR spectra clearly indicating the synthesis of the target PU(P‐Van‐N) and its three controls, namely PU(Reduced), PU(P‐N) and PU(Van‐N) (see Figure S6b,c, Supporting Information).^[^ ^24^, ^25^ ^]^ Their chemical structures are further confirmed by the corresponding ^1^H NMR spectra (Figure S7, Supporting Information). All the as‐synthesized PU elastomers show weight‐averaged molecular weights (*M*_n_) in the range of 21 to 66 kDa and relatively narrow polydispersity indexes (PDI) (<2.2) by gel permeation chromatography (GPC) (see Table S1, Supporting Information). Due to the high benzene ring content, four kinds of PU films are translucent and yellow or light yellow, in contrast to transparent and colorless PU(OH) and PU(NH_2_) (see Figure S8, Supporting Information). Interestingly, the PU(P‐Van‐N) cannot dissolve in DMSO at room temperature and only partially dissolve in THF (Figure S9, Supporting Information). Instead, it can dissolve in ethanol, methanol, *N*,*N*‐dimethylformamide (DMF) and *N*,*N*‐dimethylacetamide (DMAc), and this solubility feature makes PU(P‐Van‐N) easier to process using ethanol.

To characterize the potential *π*–*π* stacking interactions in the hard segment of PU(P‐Van‐N) as well as PU(P‐N), PU(Reduced) and PU(Van‐N), their UV–Vis spectra are recorded (Figure S10, Supporting Information). The absorption peak at around 230 nm^−1^ arises from the *π*–π* transition of the benzene rings in their structures, and the red shift for PU(P‐Van‐N) indicates a higher conjugative effect of aromatic rings in the hard segment. Meanwhile, the peak at 300 nm^−1^ is ascribed to the Schiff base C=N groups in both PU(P‐Van‐N) and PU(Van‐N), and this n‐π* transition of C=N undergoes a red shift to 400 nm^−1^, indicating that the C=N bonds can take part in the *π*–*π* stacking interactions in both PU(P‐Van‐N) and PU(Van‐N). However, the C=N bond can be readily reduced using NaBH_4_, as evidenced by the disappearance of its absorption peak in the spectrum of PU(Reduced) (Figure S10, Supporting Information).

Figure 1c schematically illustrates the *π*–*π* stacking interactions in PU(P‐Van‐N) and the stretching process. The hard phase comprising of both fire retardant groups and *π*–*π* conjugation groups are stacked within the continuous soft phase, and both *π*–*π* stacking and hydrogen‐bonding (H‐bonding) are two dominant interactions between polymer chains. The *π*–*π* stacking is further evidenced by X‐ray diffraction (XRD) results (Figure 1d), for which the C=N groups play a major role. All‐atom molecular dynamics (MD) simulations reveal that the contribution of the *π*–*π* interactions (≈104.5 kJ mol^−1^) to the total cohesive energy (402.4 kJ mol^−1^) in PU(P‐Van‐N) is as high as 26.6%.^[^ ^7^, ^26^ ^]^ (Figure 1c). By contrast, the contribution of the *π*–*π* interactions in PU(Van‐N) is lower, followed by PU(Reduced) and then PU(P‐N). In terms of H‐bonding, the numbers of H‐bonds in both PU(Reduced) and PU(P‐N) are higher than that of both PU(P‐Van‐N) and PU(Van‐N) (Tables S2 and S3, Supporting Information). The simulation results are further confirmed by their FTIR spectra by deconvoluting the C=O absorption bands.^[^ ^27^ ^]^ The fractions of H‐bonded C=O in PU(Reduced) (80.5%) and PU(P‐N) (83.7%) are much higher than that in PU(P‐Van‐N) (74.0%) (Figure S11 and Table S5, Supporting Information). This means that *π*–*π* stacking interactions are dominant interactions in both PU(P‐Van‐N) and PU(Van‐N), whereas H‐bonding dominates in both PU(Reduced) and PU(P‐N).

To determine how significantly *π*–*π* stacking and H‐bonding interactions affect the chain movement of these elastomers, the creep behaviors are carried out on PU(P‐Van‐N) and other control elastomers (see Figure 1e). PU(P‐Van‐N) first experiences a slow increase in the strain and levels off, resulting in a long‐term plateau, indicative of the presence of physical cross‐linking networks.^[^ ^28^ ^]^ By comparing with other three systems, it is the *π*–*π* stacking that determines the formation of a cross‐linking network which is further strengthened by the H‐bonding. This can be reflected by their activation energy (*E*_a_) (Figures S12 and S13, Supporting Information), which follows the order: PU(P‐Van‐N) > PU(Van‐N) > PU(Reduced) > PU(P‐N). Atomic force microscopy (AFM) is further used to observe microphase‐separated structures in PU(P‐Van‐N) and other controls (Figure 1f–i and Figures S14 and S15, Supporting Information).^[^ ^3^, ^29^, ^30^ ^]^ The PU(P‐Van‐N) exhibits worm‐like microphase separated domains (hard segments) in the order of ≈100 nm in width dispersed in the continuous matrix (soft segments). Upon chemical reduction, the phase size of hard segments PU(Reduced) significantly increases to ≈400 nm and the morphology changed to island structure. Likewise, the PU(Van‐N) displays a microphase separated morphology with well‐defined boarders. In comparison, no microphase separation is determined in PU(OH), PU(NH_2_), and PU(P‐N). Above results clearly show that the *π*–*π* stacking between hard segments can act as crosslinkers of soft segments and induce the formation of a physical crosslinking network in PU(P‐Van‐N).^[^ ^31^ ^]^

For this reason, it is expected that the dynamic *π*–*π* interactions‐induced microphase‐separated domains can lead to a large entropic elasticity of polymer chains, and thus large deformability and stretchability. Meanwhile, it also facilitates the strain‐induced crystallization of soft segments at large strains, which can result in a further increase in the strength (Figure 1c).

### 2.2 Exceptional Mechanical Properties

**Figure 2:**
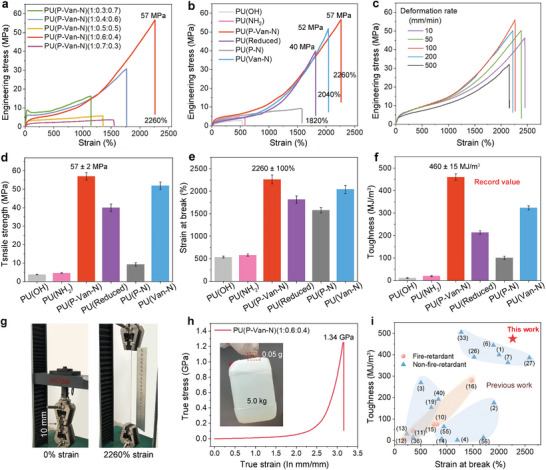
Mechanical tensile properties. Typical engineering stress–strain curves of a) the PU(P‐Van‐N) elastomers with different ratio of P‐Van‐N and PTMEG and b) the PUs with different hard segments, measured at deformation rate of 100 mm min^−1^. c) Typical engineering stress–strain curves of the PU(P‐Van‐N)(1:0.6:0.4) at various deformation rates. d) Tensile strength, e) strain at break, and f) toughness of the PUs with different hard segments. g) Photographs showing the tensile strain of the PU(P‐Van‐N)(1:0.6:0.4). h) True stress–strain curves of the PU(P‐Van‐N)(1:0.6:0.4). Inset: Photographs showing that the PU(P‐Van‐N)(1:0.6:0.4) elastomer strip (50 mg) can lift a weight of 5.0 kg. i) Comparison of the toughness and strain at break among the PU(P‐Van‐N) elastomer and some of the literature‐reported PU elastomers, including the fire retardant and none fire retardant ones. The numbers represent the reference number.

The mechanical properties of PU(P‐Van‐N) vary significantly depending on the fractions of P‐Van‐N hard segments. An increase in P‐Van‐N content leads to an improvement in both break strain and tensile strength (**Figure** **2**a). For example, PU(P‐Van‐N)(1:0.7:0.3) with a relatively high amount of PTMEG displays a very low mechanical strength (only 3.7 MPa). Upon the increase in the P‐Van‐N units to 20 mol%, that is, PU(P‐Van‐N)(1:0.6:0.4), its tensile stress and break strain sharply increase to ≈57 MPa and 2260%, respectively. With a further increase of P‐Van‐N content, the mechanical properties reduce to a different extent because a higher fraction of rigid P‐Van‐N moiety in the chain reduces the deformation ability. Interestingly, PU(P‐Van‐N)(1:0.4:0.6) shows slightly better mechanical properties than PU(P‐Van‐N)(1:0.5:0.5) owing to its microphase separated structure (Figures S14 and S15, Supporting Information). Hence, the mechanical properties of PU elastomers can be readily tuned by the content of P‐Van‐N segments.

We further characterize the mechanical properties of PUs elastomers with different hard segments at the same ratio of hard/soft segment (0.6/0.4) (Figure 2b). For both PU(OH) and PU(NH_2_), they are mechanically weak and only display a low tensile strength of less than 5.0 MPa, owing to poor interchain interactions and the lack of rigid groups in their structures. Among the other four systems, the PU(P‐Van‐N)(1:0.6:0.4) exhibits the best mechanical properties, as reflected by a high strength as high as 57 MPa, a large strain at break of 2260%, and a record high toughness of 460 MJ m^−3^ (up to 475 MJ m^−3^, see Figure 2d–g). Interestingly, its mechanical properties show a strong dependence on the stretching speed and the highest strength is achieved at a stretching rate of 100 mm min^−1^ in a stretching rate range from 10 to 500 mm min^−1^ (see Figure 2c). This is probably because the rate for the sliding and/or rupture of *π*–*π* stacking just catches up with that of external stretching (100 mm min^−1^). We also calculate the true stress–strain curve of the PU(P‐Van‐N)(1:0.6:0.4) because it can provide a more accurate indication of the capacity to withstand external stress for the highly extensible materials with large restorable deformation, because the cross‐sectional areas of such materials are dramatically decreased at break. The PU(P‐Van‐N)(1:0.6:0.4) elastomer exhibits an unprecedented true stress at break of ≈1.34 GPa (Figure 2h and Figure S16, Supporting Information), exceeding 1.21 GPa of IPDI‐SPU2000 in previous work,^[^ ^26^ ^]^ which can also be visually reflected by a thin strip of PU(P‐Van‐N) (50 mg, 0.32 mm thick, 4.0 mm wide) that can lift a weight (5.0 kg) 100 000 times its own weight without break (inset of Figure 2h).

In comparison, PU(Van‐N) is slightly weaker than PU(P‐Van‐N) thanks to the presence of *π*–*π* stacking (Figure 1d). Upon the chemical reduction of C=N bonds in PU(P‐Van‐N) to form PU(Reduced), the *π*–*π* stacking interactions weaken, thus leading to relatively lower strength (40 MPa) and strain at break (1820%), despite a moderate increase in H‐bonding. PU(P‐N) has the strongest H‐bonding interactions but relatively weaker interchain *π*–*π* stacking among these four PUs, giving rise to the worst mechanical properties. Therefore, both C=N and benzene rings in P‐Van‐N of PU(P‐Van‐N) contribute to a strong intermolecular *π*–*π* stacking that leads to high strength, large stretchability, and great toughness. Moreover, the *π*–*π* stacking plays a more important role than H‐bonding in determining the mechanical properties of these PU elastomers.

In addition, as‐designed PU(P‐Van‐N) elastomer exhibits significant hysteresis, indicative of its strong ability to dissipate fracture energy (Figure S17, Supporting Information). Meanwhile, its tensile testing in the second cycle is more compliant than that in the first cycle, indicating the chain sliding in the *π*–*π* stacking‐induced supramolecular network mainly occurring in the first cycle.^[^ ^6^, ^14^ ^]^ Moreover, PU(P‐Van‐N) also shows the best elasticity or recovery ability whereas the PU(P‐N) gives rise to the poorest recovery ability during multistep cyclic stress–strain testing (Figure S18, Supporting Information). The loading–unloading cyclic testing can be used for assessing the long‐term network stability of a material under a certain load.^[^ ^32^ ^]^ Due to the dynamic *π*–*π* stacking interactions, the PU(P‐Van‐N) presents slightly smaller hysteresis energies (*U*_hys_) than other systems at a strain below 800%, further confirming that it primarily undergoes elastic deformation (Figure S19, Supporting Information). The excellent entropic elasticity of PU(P‐Van‐N) is mainly because the hard segments with strong *π*–*π* interactions can act as physical crosslinking sites for the amorphous soft segments.

In addition, thanks to its strong ability to dissipate fracture energy, PU(P‐Van‐N) elastomer exhibits a superb crack tolerance, giving a fracture energy as large as 173.3 kJ m^−2^ (Figure S20a, Supporting Information), which is only behind the record value, 215.2 kJ m^−2^, of the IPDI‐SPU2000.^[^ ^26^ ^]^ Comparatively, PU(Van‐N) shows a lower fracture energy of 127.4 kJ m^−2^ followed by PU(Reduced) and PU(P‐N) (Figure S20b–d, Supporting Information). The results mean that the high fracture tolerance of the PU(P‐Van‐N) is most likely ascribed to the dynamic rupture and reformation of the *π*–*π* stacking in the high‐density hard domains, resulting in the stick–slip motion of polymer chains upon external forces. Consequently, stress concentrated at the crack tip can be effectively transferred to the entire polymer network, thus prohibiting the lateral propagation of the crack.

The mechanical properties of as‐developed PU(P‐Van‐N)(1:0.6:0.4) are also compared with previous PU elastomers (Figure 2i and Table S6, Supporting Information). It is worth noting that as‐developed PU(P‐Van‐N) is inherently fire retardant and possesses a self‐extinguishing ability, which will be discussed in the following section. On the whole, PU(P‐Van‐N) exhibits the best combination of fracture toughness (≈460 MJ m^−3^), break strain (≈2260%) and strength (≈57 MPa), superior to any of its previous counterparts, and also hitting a record high for all existing fire retardant PU elastomers known to date. For instance, despite a comparable break strain (≈2000%), the PU elastomer, PU‐UPy‐DOPA‐Fe^[^ ^6^ ^]^ exhibits a lower toughness of ca. 405 MJ m^−3^ and a lower tensile strength of 47 MPa (Figure 2i and Table S6, Supporting Information). Similarly, the 0.5%‐AD/GO‐PU shows a relatively low break strain of ≈1273% although it has very high toughness (505.7 MJ m^−3^) and tensile strength (78.3 MPa).^[^ ^33^ ^]^ Another example is the PT‐HM‐U20 elastomer, which shows a higher break strain (≈2580%) but smaller strength (48.5 MPa) and toughness (≈386.5 MJ m^−3^),^[^ ^27^ ^]^ respectively, 15% and 16% lower than the corresponding value of the PU(P‐Van‐N). Furthermore, both PUs do not have a self‐extinguishing ability, which can limit their practical applications in the field, for example, electronics, where a demanding fire retardancy is often required. On the other hand, for fire retardant PU elastomers, although the reported PEPE‐TPU^[^ ^16^ ^]^ also shows a desirable fire retardance, it exhibits moderately lower stretchability (1470%), strength (39.2 MPa) and toughness (≈280 MJ m^−3^), all of which are inferior to those of PU(P‐Van‐N). These comparisons indicate that as‐designed PU(P‐Van‐N) exhibits a superior performance portfolio in terms of toughness and intrinsic fire retardancy.

### 2.3 Mechanical Mechanisms

#### 2.3.1 Mechanical Mechanisms by Experiment

**Figure 3:**
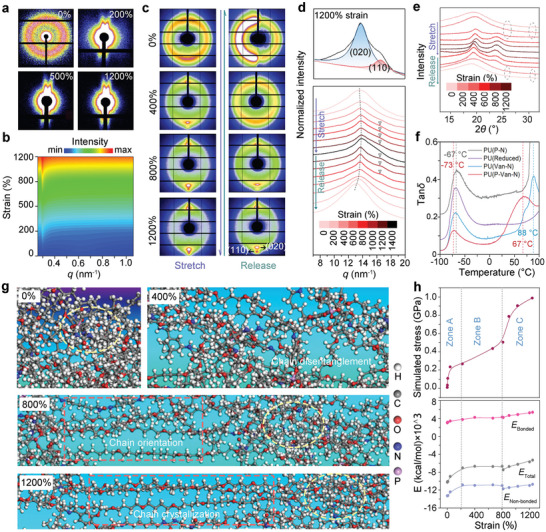
Mechanical mechanisms by experiment and molecular dynamic (MD) simulation. a) 2D SAXS patterns and b) the corresponding contour maps of the PU(P‐Van‐N) at various strains. c) 2D WAXD patterns, d) 1D WAXD profiles, and e) XRD patterns of the PU(P‐Van‐N) at different strains. f) Temperature dependence of the tan*δ* for PU(P‐Van‐N), PU(Reduced), PU(P‐N), and PU(Van‐N). g) Snapshots showing the MD simulations of the PU(P‐Van‐N) at strains of 0%, 400%, 800%, 1200%. h) Simulated stress–strain curves of PU(P‐Van‐N) samples (Zone A: chain entanglement; Zone B: breakage of *π*–*π* bonding; Zone C: chain orientation and crystallization) and corresponding total, bonded, and non‐bonded energy during stretching of PU(P‐Van‐N) based on MD simulation.

2D small‐angle X‐ray scattering (2D SAXS) is first employed to characterize the structural changes in the dynamic hierarchical domains of PU(P‐Van‐N) during stretching.^[^ ^2^, ^5^, ^6^, ^33^, ^34^ ^]^ The 2D SAXS pattern shows a circular ring before stretching, indicating isotropically orientated P‐Van‐N domains (**Figure** **3**a). Upon stretching, the patterns clearly exhibit the growing deformation of phase domains, as reflected by deformed scattering circles, suggesting the steady alignment of the domains in the stretching direction. It is further confirmed by their corresponding contour maps (Figure 3b and Figure S21a, Supporting Information).^[^ ^36^ ^]^ To quantify the change of domains, we extract the radius of gyration (*R*_g_) and the distance between two neighboring phases (*R*_d_) from the 1D SAXS spectra (Table S7, Supporting Information).^[^ ^26^ ^]^ Before stretching, the *R*_g_ and *R*_d_ of PU(P‐Van‐N) are calculated to be 2.7 and 10 nm. Upon stretching to 200%, the *R*_g_ and *R*_d_, respectively, increase to 6.0 and 21.7 nm, due to the affine deformation of the soft segments and straightening of the chains between conjunction points. With further stretching, there are no further determined changes in both *R*_g_ and *R*_d_ values, suggesting that the hard segments tend to slide and undergo a dynamic change of *π*–*π* stacking at this stage. The dynamic domains can also facilitate the absorption and dissipation of fracture energy, in addition to the deformation of the *π*–*π* stacking‐crosslinked soft segments.

The above results are further confirmed by 2D wide‐angle X‐ray diffraction (WAXD) that gives the intensity distribution profiles of PU elastomers.^[^ ^37^, ^38^ ^]^ Prior to stretching, there is only an isotropic halo in its WAXD patterns, indicating that polymer chains are in an equilibrium amorphous state and no crystal is determined (Figure 3c). Upon being stretched to 400%, the amorphous halos begin to retrograde and crystal diffraction arcs gradually appear. With a further increase in strain, the crystal diffraction become increasingly strong, manifesting the continuous enhancement of orientation of soft segments and then induced crystallization. Due to this strain‐induced orientation, the diffraction patterns of PU(P‐Van‐N) only show a partial recovery after unloading. Meanwhile, the two diffraction spots observed in the 2D WAXD patterns correspond to the deconvoluted Peak 1 and Peak 2 in the 1D WAXD profiles, centered at the scattering vector *q* of 13.6 and 16.5 nm^−1^ (Figure 3d), respectively, assigned to the (020) and (110) planes of the crystalline methylene groups in the PTMEG segments.^[^ ^37^, ^38^, ^39^ ^]^ The newly‐formed PTMEG crystalline regions can also serve as physical crosslinking sites to form a self‐reinforcing phase, thus leading to a sharp increase in mechanical properties of PU(P‐Van‐N), as evidenced by a strain‐hardening zone (Figure 2a).

The strain‐induced crystallization behavior of PU(P‐Van‐N) is further evidenced by in‐situ XRD (Figure 3e). Upon being stretched to 400%, two new peaks centered at 19.8° and 23.9° appear, respectively, corresponding to the *d*‐spacing of 0.45 and 0.37 nm, which are very close to the *d*‐spacing (*d* = 2*π*/*q*_max_) of 0.46 and 0.38 nm obtained by WAXD testing. The in‐situ XRD gives rise to more significant crystallization behaviors mainly because of the greater waiting time allowed for the stretching sample to crystallize, rather than only a 10 s exposure in 2D WAXD testing. Furthermore, the intensities of two peaks at 25.9° and 31.0° belonging to the *π*–*π* stacking in the hard segments of PU(P‐Van‐N) (see Figure 1d) gradually weaken with stretching and completely disappear at 600% strain. These two peaks reappear with the release of external load. This further verifies that *π*–*π* stacking‐induced cross‐linking points are dynamic and fully reversible, and this feature enables high strength, large stretchability and great toughness of PU(P‐Van‐N) by resisting the external force and rapidly dissipating the energy at high strains.

In addition, the PU(P‐Van‐N) shows two distinct glass transition temperatures (*T*_g_), located at −73 and 67 °C (Figure 3f), which, respectively, correspond to the glass transition of the soft PTMEG segments and hard segments. However, once PU(P‐Van‐N) is chemically reduced, the higher *T*_g_ disappears, indicating this glass transition arises from the *π*–*π* stacking of P‐Van‐N motifs, as evidenced by a comparable high *T*_g_ in PU(Van‐N). Because of the good compatibility between hard and soft segments, both PU(Reduced) and PU(P‐N) do not show the glass transition of hard segments in this temperature range, which is in good accordance with atomic force microscopy (AFM) results (Figure 1). In brief, the lower *T*_g_ of soft segments in PU(P‐Van‐N) provides the elastomer a wide elastic range, whereas the high *T*_g_ of *π*–*π* stacking‐induced hard segments enables high strength.^[^ ^40^ ^]^

#### 2.3.2 Mechanical Mechanisms by MD Simulation

Molecular dynamics (MD) simulations are also used to gain an understanding of the unique combination of high strength, large stretchability, and great toughness in PU(P‐Van‐N) on the molecular scale.^[^ ^41^, ^42^, ^43^ ^]^ The PU(P‐Van‐N) chains are first entangled at lower strains and then orientate along with the chain disentanglement upon stretching (Figure 3g and Movie S1, Supporting Information). During stretching, the *π*–*π* stacking experiences fast break and reforming (ellipse marked in Figure 3g), leading to the gradual orientation of PU(P‐Van‐N) chains. When the strain exceeds 800%, the alignment of polymer chains makes it possible for the soft PTMEG segments to crystallize. This strain‐induced crystallization then results in strain‐hardening that can significantly increase the mechanical strength, yielding a simulated stress of about 1.0 GPa comparable to the true stress, 1.34 GPa (Figure 2h).

The MD simulation presents that the stretching of PU(P‐Van‐N) undergoes three phases, as indicated in Zones A–C (Figure 3h). At small strains (<200%, Zone A), the soft segments in the elastomer tend to disentangle and extend, whereas the phase‐separated hard domains maintain the structural integrity. At this stage, both the non‐bonded and total energy decrease, leading to slow increases in both modulus and strength. With further stretching (strains > 200%, Zone B), the PTMEG segments gradually align in the stretching direction, whereas the *π*–*π* stacking interactions in the hard segments experience repeated disassociation and association, giving rise to a steady increase in the deformation of the elastomer. Due to the dynamic nature of *π*–*π* interactions in hard segments, the total energy stays steady in this phase. At large strains, >800% (Zone C), the strain induces the nucleation and crystallization PTMEG moieties, which substantially increases the energy barrier for the deformation and rupture of the elastomers. As a result, the PU(P‐Van‐N) shows dramatic increases in strength, stretchability and toughness, as evidenced by the increased bond energy and bond angle energy.

In brief, the combination of high strength, high ductility, and great toughness in the PU(P‐Van‐N) mainly originates from the dynamic *π*–*π* stacking interactions of P‐Van‐N motifs in the chain backbones and the strain‐hardening effect as a result of the strain‐induced crystallization of PTMEG segments. Interestingly, a similar phenomenon can also be observed in PU(Van‐N) but not in both PU(P‐N) and PU(Reduced) (Figure 3g and Figures S23–S25, Supporting Information). This further implies that the Schiff base (C=N groups)‐induced *π*–*π* stacking is very effective in enhancing the overall mechanical properties of elastomers.

### 2.4 Exceptional Intrinsic Fire Retardancy

The P‐Van‐N motif is rationally designed to integrate fire retardant groups: P‐containing units and C=N bonds. The P‐containing moiety can interrupt the combustion via capturing the free radicals in the gas phase, and catalyze the char formation in the condensed phase.^[^ ^44^, ^45^ ^]^ The C=N bonds can form thermostable cross‐linking networks through self‐crosslinking at high temperatures.^[^ ^46^, ^47^, ^48^ ^]^ In addition, *π*–*π* stacking interactions are expected to increase the char residue and thus improve the fire retardancy.

**Figure 4:**
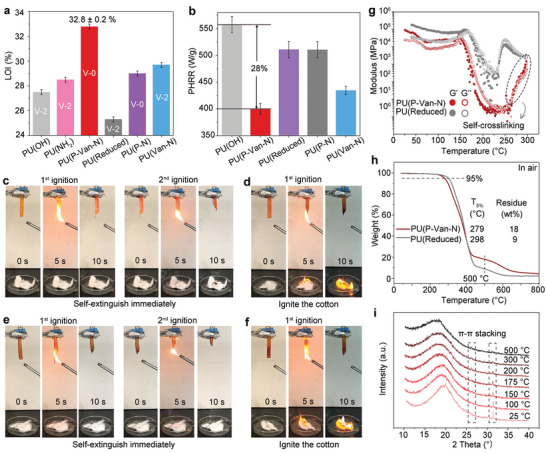
Intrinsic fire retardancy. a) LOI and UL‐94 results and b) PHRR values for PU elastomers. UL‐94 test for c) PU(P‐Van‐N), d) PU(Reduced), e) PU(P‐N), and f) PU(Van‐N) respectively. g) Storage modulus (G′) and loss modulus (G″) of the PU(P‐Van‐N) and PU(Reduced) as a function of temperature. h) TGA curves for PU(P‐Van‐N) and PU(Reduced) in air atmosphere. i) XRD patterns of PU(P‐Van‐N) during the heating process.

The PU(P‐Van‐N) exhibits a high LOI value of 32.8%, far greater than that of the other four PU elastomers (**Figure** **4**a and Table S8, Supporting Information). This indicates a strong additive effect between P‐containing units, C=N groups, and *π*–*π* stacking interactions in terms of promoting LOI values. Meanwhile, microscale cone calorimetry (MCC) is also employed to further evaluate the fire performance of PU elastomers.^[^ ^45^, ^49^ ^]^ The PU(P‐Van‐N) presents the lowest peak of heat release rate (PHRR) value among all systems, manifesting its best fire performance, which is in good accordance to the LOI results (Figure 4b and Table S8, Supporting Information). Moreover, the PU(Van‐N) shows a slightly higher peak of heat release rate (PHRR) value than PU(P‐Van‐N), which means that C=N groups play a key role in reducing the heat release of the elastomers. Vertical burning testing is extensively applied to assess the fire retardant levels of a material in industries. Both PU(P‐Van‐N) and PU(P‐N) show an ability to self‐extinguish and to pass the desired UL‐94 V‐0 rating, but other three PU systems do not (Figure 4c–f), clearly indicative of the key role of P‐containing moiety in fire retardancy of the elastomers.^[^ ^50^ ^]^ Therefore, the combination of P‐containing groups and C=N bonds as well as the *π*–*π* stacking contribute to the exceptional intrinsic flame retardancy of PU(P‐Van‐N).

### 2.5 Fire Retardant Mechanism

It is essential to verify our hypothesis regarding the contributions of three key moieties to the excellent fire retardancy of PU(P‐Van‐N). Before 230 °C, the PU(P‐Van‐N) shows moderately lower storage modulus and loss modulus than PU(Reduced) due to thermally reversible C=N bonds in the PU(P‐Van‐N) (Figure 4g).^[^ ^51^ ^]^ However, once the temperature exceeds 350 °C, the modulus of PU(P‐Van‐N) sharply increase because of self‐crosslinking of C=N bonds and polymer chains at high temperatures,^[^ ^46^, ^47^, ^48^ ^]^ which can promote the formation of thermostable char residues. As a result, the PU(P‐Van‐N) yields more char residues at 500 °C, twice that of PU(Reduced) for which its char is mainly created by the P‐containing units (Figure 4h), and its char has a higher degree of graphitization than that of the latter (Figure S26, Supporting Information).^[^ ^44^ ^]^ Moreover, the PU(P‐Van‐N) undergoes a lower degree of oxidation than PU(Reduced) during heating (Figure S27c, Supporting Information), which also contributes to the good fire retardancy of PU(P‐Van‐N) by increasing the formation of thermostable char. In addition, the *π*–*π* stacking can still be determined in PU(P‐Van‐N) even at elevated temperatures (Figure 4i), which can improve the structural integrity of the residual char (Figure S28, Supporting Information), thus contributing to the fire retardancy of PU(P‐Van‐N).^[^ ^52^, ^53^ ^]^ In comparison, the interactions cannot be found in the XRD pattern of PU(Reduced) (Figure S29, Supporting Information). Therefore, it is the combination of the catalytic carbonization of P‐containing units, the cyclizing of C=N and the *π*–*π* stacking, that contribute to the self‐extinguishing ability of PU(P‐Van‐N).

### 2.6 Chemical Degradability

The dynamic nature of C=N bonds allows for dissociation of PU(P‐Van‐N) under alkaline or acid conditions.^[^ ^54^, ^55^ ^]^ After the film is immersed in a 5 wt% aqueous solution of acetic acid, its color gradually turns from red to white, implying the hydrolysis of the C=N bonds. However, the degraded PU(P‐Van‐N) film does not dissolve in the solvent due to its poor solubility in water (Figure S30, Supporting Information). Due to its good solubility in ethanol, the PU(P‐Van‐N) film can readily degrade in an ethanol solution of acetic acid (Figure S30, Supporting Information). In the presence of acetic acid, C=N experiences chemical cleavages, as evidenced by its degraded fragments (Figure S31, Supporting Information), all of which can completely dissolve in ethanol. Moreover, the degraded PU(P‐Van‐N) can be remolded by evaporating the solvent. Interestingly, the chemical structure of recycled PU(P‐Van‐N) does not show significant changes (Figure S32a, Supporting Information). However, its molecular weight decreases from 65 700 to 40 300 g mol^−1^ (Figure S30, Supporting Information), and the mechanical properties also slightly decrease (Figure S32b, Supporting Information). Therefore, the as‐developed PU(P‐Van‐N) elastomers can be chemically degraded in a non‐toxic solvent and also be recycled without deteriorating its mechanical properties.

### 2.7 PU(P‐Van‐N) as a Reliable Substrate for Strain Sensors

**Figure 5:**
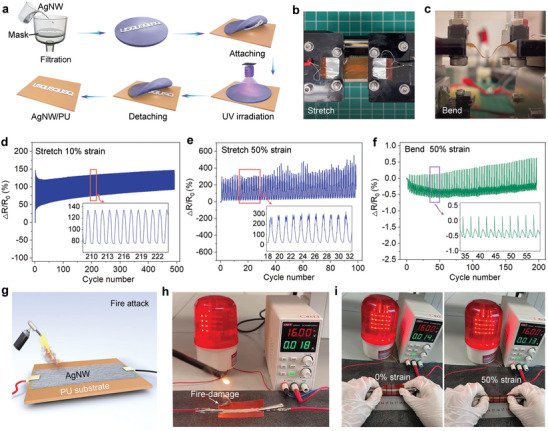
PU(P‐Van‐N) as a reliable substrate for flexible strain sensors under stretching, bending, and fire attacks. a) Schematic representation of the fabrication steps of AgNW/PU. Photographs of AgNW/PU at b) stretching and c) bending process. d) Transient resistance measured over 500 cycles of stretching–relaxation between 0% and 10% strain. e) Transient resistance measured over 100 cycles of stretching–relaxation between 0% and 50% strain. f) Transient resistance measured over 200 cycles of bending–relaxation between 10 and 5 mm. g) Schematic representation of the fire attack of AgNW/PU film. The electrical conductivity of AgNW/PU film h) when ignited and i) after igniting at strain of 0% and 50%.

We investigate the potential application of as‐developed PU(P‐Van‐N) elastomer as a flexible film substrate for strain sensors.^[^ ^56^ ^]^ The strain sensors can be readily fabricated by depositing Ag nanowires (AgNW) onto its surface (**Figure** **5**a and Figure S33, Supporting Information) and electrical signals will be recorded in response to stretching and bending (Figure 5b,c and Movies S2 and S3, Supporting Information).^[^ ^33^, ^57^, ^58^ ^]^ The strain sensor shows a high sensitivity to strain and maintains a stable response over 500 cycles of stretching–relaxation between 0 and 10% strain. The electrical resistance change (*∆R*/*R*_0_) presents an instantaneous response to the change in strain and a good reproducibility over the loading–unloading cycles from 210 to 222 cycles (Figure 5d), indicating its reliable sensing performance. When the stretching–relaxation is carried out between 0 and 50% strain, the AgNW/PU sensor can still work in a stable manner for over 100 cycles, and the overall *∆R*/*R*_0_ is much higher, suggesting its strong strain dependence of electrical resistance (Figure 5e). Interestingly, this strain sensor is insensitive to bending, as reflected by a very small resistance change less than 0.5%.^[^ ^59^, ^60^ ^]^ The high sensitivity to stretching but insensitivity to bending makes the strain sensor a reliable sensor for stretching.

In addition, it is desirable for strain sensors, such as the electronic skin of robots, to possess intrinsic fire retardancy for their real‐world applications (Movie S4, Supporting Information). Upon exposure to a fire attack, the AgNW/PU electronic skin (Figure 5g) is expected to show an ability to extinguish flame immediately. When the sensor is ignited by a butane torch, it can self‐extinguish instantly, and thus the sensor can stay running even when the fire‐damaged sensor is stretched up to 50% (Figure 5h,i and Movie S5, Supporting Information), indicating its high reliability when exposed to external fire attacks. These applications clearly demonstrate that as‐developed PU elastomers hold great promise for flexible electronics and devices applications.

### 2.8 Ideal Recyclability of AgNW/PU Strain Sensors

It is highly desirable to recycle expensive AgNW from the waste sensors under a mild condition. Unfortunately, most of existing flexible strain sensors often suffers from the difficulty in their recycling due to the poor solubility of soft polymer substrates in common solvents, such as ethanol. Because of the excellent solubility of PU(P‐Van‐N) in ethanol, the AgNW can be easily recycled from the AgNW/PU sensors using ethanol. The recovery rate of AgNW exceeds 90% (Figure S34, Supporting Information), with slight changes in its morphology (Figure S35, Supporting Information). Moreover, the recycled PU(P‐Van‐N) elastomer can nearly preserve its excellent mechanical properties (Figure S36, Supporting Information). The results strongly show that as‐created PU(P‐Van‐N) elastomer can be an ideal reliable yet recyclable substrate for soft electronics and devices.

### 2.9 PU(P‐Van‐N) as a Recyclable Binder of Si/C Anodes

In addition, we also examine the potential application of PU(P‐Van‐N) as a binder for Si/C anode of Li‐ion batteries. The results clearly demonstrate that as‐developed PU(P‐Van‐N) can be a comparable yet recyclable binder for Si/C anode, even when exposed to fire/flame (Figures S37 and S38, and Movie S6, Supporting Information).^[^ ^61^, ^62^, ^63^, ^64^, ^65^, ^66^, ^67^ ^]^

## 3 Conclusion

In summary, we successfully fabricated highly stretchable, ultratough yet strong PU(P‐Van‐N) elastomers with intrinsic fire retardancy and recyclability by the inclusion of dynamic C=N bonds and phosphorus‐containing moieties. The PU elastomer exhibits a superhigh true stress at break of 1.34 GPa, the highest reported toughness to date of 460 MJ m^−3^, and a large break strain of ≈2260%, exceeding all reported PU elastomers. Moreover, it exhibits an intrinsic fire retardancy, with an LOI value as high as 32.8% and a UL‐94 V‐0 rating. The exceptional mechanical properties are primarily attributed to the well‐designed dynamic *π*–*π* stacking in the hard segments that can act as crosslinkers to increase the entropic elasticity, and the strain‐hardening as a result of the strain‐induced crystallization of soft segments. Its superior fire retardancy is ascribed to the combined effect of the catalytic charring effect of phosphorus‐containing units, the cyclization of C=N at high temperatures and the char promotion effect of *π*–*π* stacking interactions. Because of its fire retardancy and good ethanol‐solubility, the PU elastomers show great potential as a reliable and recyclable substrate for strain sensors as well as a recyclable binder for Si/C anode. This work offers a new design approach for next‐generation sustainable advanced soft materials for flexible electronic device and lithium‐ion battery applications.

## Conflict of Interest

The authors declare no conflict of interest.

## Supporting information

Supporting Information

Supplemental Movie 1

Supplemental Movie 2

Supplemental Movie 3

Supplemental Movie 4

Supplemental Movie 5

Supplemental Movie 6

## Data Availability

Research data are not shared.
