# Supplementary material for: Stretchable, Ultratough, and Intrinsically Self‐Extinguishing Elastomers with Desirable Recyclability
Source: Adv Sci (Weinh). 2023 Jan 22;10(9):2207268. doi: 10.1002/advs.202207268 (PMC10037964; doi:10.1002/advs.202207268)
Supplement: Supplementary file 1 — Supporting Information [file ADVS-10-2207268-s003.pdf]

Supplementary Information

Stretchable, ultratough and intrinsically self-extinguishing elastomers with desirable recyclability

Yijiao Xue, Jinyou Lin, Tao Wan, Yanlong Luo, Zhewen Ma, Yonghong Zhou, Bryan T. Tuten, Meng Zhang, Xinyong Tao and Pingan Song**

Y. J. Xue, Y. H. Zhou, M. Zhang
Institute of Chemical Industry of Forest Products, Chinese Academy of Forestry (CAF)
Nanjing, 210042, China
E-mail: zhangmeng@icifp.cn (M. Zhang)

J. Y. Lin
Shanghai Advanced Research Institute, Chinese Academy of Sciences
Shanghai, 201204, China

T. Wan
School of Materials Science and Engineering, The University of New South Wales
Sydney, NSW 2502, Australia

Y. L. Luo
College of Science, Nanjing Forestry University
Nanjing, 210037, China

Z. W. Ma
Department of Polymer Materials, School of Materials Science and Engineering, Tongji University
Shanghai, 201804, China

B. T. Tuten
Centre for Materials Science, School of Chemistry and Physics, Queensland University of Technology
Brisbane, QLD 4000, Australia

X. Y. Tao
College of Materials Science and Engineering
Zhejiang University of Technology
Hangzhou, 310014, China

P. A. Song
Centre for Future Materials, Unviersity of Southern Queensland
Springfield 4300, Australia

School of Agriculture and Environmental Science, Unviersity of Southern Queensland
Springfield 4300, Australia
E-mail: Pingan.Song@usq.edu.au

Table of contents

Experimental Section
Characterizations
Figure S1-S38
Table S1-S8
Movies S1-S6
References

1. Experimental Section

1.1. Materials.

Polytetramethylene ether glycol (PTMEG, M_n = 1000 g/mol) and vanillin (Van, 99%) were purchased from Rhawn. Isophorone diisocyanate (IPDI, 99%), phenylphosphonic dichloride (PPDC, 98%), N,N-dimethylacetamide (DMAc, 99.8%), ethanol (99%), 1,4-Dioxane (99%), tetrahydrofuran (THF, 99%), Triethylamine (TEA, 99%), 1,6-hexamethylenediamine (HDI, 98%) and 1,6-hexanediol (99%) were purchased from Macklin. Dibutyltin dilaurate (DBTDL, 95%), Toluene (99.5%) and sodium borohydride (NaBH4, 98%) were purchased from Sigma-Aldrich.

1.2. Synthesis of P-Van-N

The synthesis of P-Van-N was conducted in a 100 mL four-neck round-bottom flask equipped with a mechanical stirrer, thermometer, and condenser. The flask was charged with 50 g THF, Van (9.22 g, 0.06 mol) and TEA (6.13 g, 0.06 mol) at 0°C. PPDC (6.57 g, 0.03 mol) in 10 g THF were added dropwise to the mixture in the mechanically stirred flask over a period of 0.5 h. Subsequently, the mixture was stirred for another 2 h at 0°C and then 6 h at 30°C. After that, the intermediate products were obtained by extraction filtration and evaporation of THF. (13.10 g, yield = 89.3%). Next, the intermediate products in 50 ml ethanol and HDI (7.04 g, 0.06 mol) in 50 ml deionized water were mixed in a 250 mL three-necked flask. The mixed solution was stirred and then heated at 40°C for 4 h. Subsequently, the reaction system was cooled down (25°C) and then filtered, and the filtration residue was washed three times using ethanol and deionized water respectively. After that, the product was dried at 100°C for 24 h, and

the rufous solid P-Van-N was obtained (18.34 g, yield = 93.7%). The synthetic route for P-Van-N is shown in Figure 1a.

1.3. Synthesis of P-N

The synthesis of P-N was conducted in a 100 mL four-neck round-bottom flask equipped with a mechanical stirrer, thermometer, and condenser. The flask was charged with 50 g 1,4-Dioxane, HDI (7.04 g, 0.06 mol) and TEA (6.13 g, 0.06 mol) at 0°C. PPDC (6.57 g, 0.03 mol) in 10 g 1,4-Dioxane were added dropwise to the mixture in the mechanically stirred flask over a period of 0.5 h. Subsequently, the mixture was stirred for another 2 h at 60°C. After that, the products were obtained by extraction filtration and evaporation of 1,4-Dioxane (10.92 g, yield = 87.5%). The synthetic route for P-N is shown in Figure S1, Supporting Information.

1.4. Synthesis of Van-N

Van (6.09 g, 0.04 mol) in 50 ml ethanol and HDI (2.32 g, 0.02 mol) in 50 ml deionized water were mixed in a 250 mL three-necked flask. The mixed solution was stirred and then heated at 40°C for 4 h. Subsequently, the reaction system was cooled down (25°C) and then filtered, and the filtration residue was washed three times using ethanol and deionized water respectively. After that, the product was dried at 100°C for 24 h, and the yellow solid Van-N was obtained (7.37 g, yield = 95.6%). The synthetic route for Van-N is shown in Figure S2, Supporting Information.

1.5. Synthesis of PU elastomers

The typical synthetic routes for PU(P-Van-N)(1:0.6:0.4) are shown in Figure 1a and described in detail as follows. Firstly, a three-neck round bottom flask equipped with a mechanical stirrer was charged with PTMEG (M_n of 1000 g mol^{-1}, 3 mmol), followed by heating at 110°C under vacuum and stirring for 30 min to remove the moisture. After cooling the reaction system to 80°C, the mixture of IPDI (5 mmol) and dibutyltin dilaurate (DBTDL, 0.02 g) dissolved 20 mL in DMAc was added into the flask. The reaction system was further heated at 80°C under N_2 atmosphere and stirred for 3 h. Consequently, P-Van-N (2 mmol) as the chain extender, dissolved in 20 mL DMAc was added into the reaction system that was further kept at 80 °C for 3 h under stirring and N_2 atmosphere. Viscous and orange solutions of the PU(P-Van- N)(1:0.6:0.4) was finally obtained. The as-obtained polymer solutions were finally poured into PTFE plates and then dried at 80 °C overnight in a drying oven, followed by drying at 80 °C for 12 h under vacuum to obtain the elastomer samples. The films (thickness: *ca.* 300 μm) were cut into different shapes for further measurements.

Following the same procedure for PU(OH)(1:0.6:0.4), PU(NH_2)(1:0.6:0.4), PU(P-N)(1:0.6:0.4) and PU(Van-N)(1:0.6:0.4), they were synthesized by replacing P-Van-N with 1,6-hexanediol, 1,6-hexamethylenediamine, P-N and Van-N respectively. The chemical structure for PU(OH)(1:0.6:0.4) and PU(NH_2)(1:0.6:0.4) are shown in Figure S3, Supporting Information.

For the synthesis of PU(Reduced), PU(P-Van-N) (1.01g, 0.01 mol) were dissolved in a 50 mL three-necked flask containing 10 mL of toluene. $NaBH_4$ (0.15 g, 0.05 mol)

were added dropwise to the solvent in the magnetic stirred flask over a period of 4 h. After that, the products were obtained by extraction filtration and washed three times using deionized water. The precipitate was dried under vacuum at 100°C for 24 h to obtain PU(Reduced) (1.65 g, yield = 89.3%). The chemical structure for PU(Reduced) is shown in Figure 1a.

Following the same procedure as PU(P-Van-N)(1:0.6:0.4), the PU(P-Van-N)(1:0.3:0.7), PU(P-Van-N)(1:0.4:0.6), PU(P-Van-N)(1:0.5:0.5) and PU(P-Van-N)(1:0.7:0.3) were synthesized by changing the feed mass of PTMEG and P-Van-N as follows: PTMEG (1.5 mmol) and P-Van-N (3.5 mmol) for PU(P-Van-N)(1:0.3:0.7); PTMEG (2.0 mmol) and P-Van-N (3.0 mmol) for PU(P-Van-N)(1:0.4:0.6); PTMEG (2.5 mmol) and P-Van-N (2.5 mmol) for PU(P-Van-N)(1:0.5:0.5); PTMEG (3.5 mmol) and P-Van-N (1.5 mmol) for PU(P-Van-N)(1:0.7:0.3). For convenience, PU(P-Van-N) was abbreviated for PU(P-Van-N)(1:0.6:0.4) in the following text and the main manuscript.

1.6. Characterization

1.6.1. General characterization

The FTIR spectra were taken on a Nicolet iS10 IR spectrometer with the scanning range from 4000 to 400 cm^{-1}. ^{1}H-NMR spectra were carried out on a Bruker ARX 300 NMR spectrometer with $CDCl_3$ as the solvent. Molecular weights of the PUs were measured using gel permeation chromatography (GPC, Waters 1515) with tetrahydrofuran (THF) as the mobile phase at a flow rate of 1 mL min^{-1}. The sample concentration was approximately 2-3 mg mL^{-1} with an injection volume of 60 μL. UV spectra were

recorded on a Shimadzu UV-1900 UV-Vis Spectrophotometer. X-ray diffraction (XRD) measurements were carried out on an D8 FOCUS X-ray diffractometer (Bruker Co., German). XRD at different temperatures during the heating processes was conducted on a Panalytical X-ray diffraction system at a rate of 20°C min^{-1}. Thermogravimetric analyses (TGA) were performed on the TG209F1 instrument in the temperature ranging from 50 to 800°C at the heating rate of 10°C min^{-1} in air atmosphere. Atomic force microscope (AFM) was conducted on a Shimadzu SPM-9600 instrument to collect the AFM height and phase images simultaneously.

Rheological measurements were performed on an HAAKE (German) in a dynamic mode on parallel-plate geometry with a diameter of 25 mm and a gap of around 1 mm. Storage moduli (G') and loss moduli (G'') were measured in temperature dispersion tests in a range from 25 to 300°C (5°C/min) at a constant frequency of 0.1 rad/s with an applied strain (γ) of 10%. Frequency sweep tests were conducted in a range from 0.01 rad/s to 100 rad/s at designated temperatures with an γ of 10%. Dynamic mechanical analysis (DMA) was carried out using a DMA Q800 (TA) equipped with tension film clamps. The temperature ramp test was performed from -100 to 100°C, 3°C/min, at 1 Hz with an amplitude of 15 μm. Moreover, the creep measurements were performed at constant creep stress of 0.1 MPa, at 35°C. All samples were isothermal at 35°C by 15 min before the test and then creep stress was loaded by 300 min followed by 120 min recovery.

Limited oxygen index (LOI) was measured on a JF-3 oxygen index meter (Jiangning, China) with sheet dimensions of 130 × 6.5 × 0.3 mm^3 according to ISO4589-1984. The

UL-94 rating was tested according to the UL-94 (ASTM D 63577) standard. The sample has a size of 130 × 13 × 0.3 mm^3. The flammability properties of samples were evaluated using a microscale combustion calorimeter (MCC) in a govmark MCC-2 device in N_2/O_2 atmosphere. Laser Raman spectroscopy (LRS) measurements were carried out at room temperature with a LabRAM HR800 with excitation by a 325 nm helium-neon laser line focused a micrometer spot on the sample surface in a scanning range of 200-4500 cm^{-1}. Thermogravimetric analysis/infrared spectrometry (TG-IR) was carried with the same instrument above coupled with a Fourier transform infrared (FTIR, Thermo Nicolet iS10, Thermo Scientific, Germany) spectroscope. The morphology of the char residue was observed using Field emission scanning electron microscope (FEI-SEM, SU8010) at an accelerating voltage of 3 kV. The degradation products were measured by LCMS-IT-TOF (Shimadzu, Japan) with methyl alcohol and methanoic acid as solvent.

1.6.2. Tensile tests

The tensile tests were mainly evaluated *via* stress-stain curves using a CMT40005 electronic universal tensile tester at room temperature. The whole samples were cut into a dumbbell shape (a testing size of 10.0 × 4.0 × 0.3 mm^3) and tested in quintuplicate at different strain rates.

True stress (σ_t) and true strain (ε_t) were calculated based on the engineering stress-strain curves according to the following equation (S1) and (S2):

$$\sigma_t = \sigma * \frac{L}{L_0} = \sigma * (\varepsilon + 1) \qquad \text{(S1)}$$

$$\varepsilon_t = \int_{L_0}^{L} \frac{dL}{L} = ln\frac{L}{L_0} = ln(\varepsilon + 1) \quad \text{(S2)}$$

where σ is the engineering stress, L and L_0 are respectively the instant and original

Tensile toughness (τ) was determined by integrating the area under the engineering stress (σ)-strain (ε) curves according to the following equation (S3)

$$\tau = \int_{\varepsilon=0}^{\varepsilon=\varepsilon_{max}} \sigma d\varepsilon \quad \text{(S3)}$$

where σ is the engineering stress, ε is the engineering strain, ε_{max} is the strain at break.

For the cyclic tensile test, both loading and unloading processes was performed at a strain rate of 100 mm min^{-1} at room temperature. The samples were stretched to a strain of 400% for 10 times.

For the hysteresis experiment, we applied consecutive loading-unloading cycles up to strains of 100%, 200%, 400%, 600%, 800%, 1000%, 1200%, 1400% and 1600% at a rate of 100 mm min^{-1} at each step. The dissipated energy was defined as the area of the hysteresis loop encompassed by the loading-unloading curve, which was calculated by integrating the area under the stress-strain curve.

1.6.3. Measurement of the fracture energy

For fracture energy tests, pure tensile tests were performed with 5 kN load cell using the single-edge notched sample in gauge length of 10 mm, width of 4 mm, thickness of 0.3 mm and slit of 1 mm. Both notched and unnotched specimens were tested at the stretching speed of 3 mm min^{-1}. The fracture energy (G_c) was calculated by the formula:

$$G_c = \frac{6wc}{\sqrt{\lambda_c}} \quad \text{(S4)}$$

where c is the critical length of the slit, λ_c is the stretch of the crack propagation, w is the strain energy obtained by integrating the area under the stress-strain curve of the

unnotched sample at the ε_c ($\varepsilon_c = \lambda_c - 1$).[1] The results were repeated for three times on different batches of samples.

1.6.4. Small-angle X-ray scattering (SAXS) measurements

The SAXS experiments were conducted at beamline BL16B1 at the Shanghai Synchrotron Radiation Facility (SSRF). The X-ray wavelength was 0.12398 nm and the sample-to-detector distance is 2235 mm. The two-dimensional (2D) SAXS patterns was recorded within the exposure time of 15 min by a Pilatus 2M detector with 1475 × 1679 pixels (resolution: 172 × 172 μm^2). The one-dimensional SAXS curves were integrated by averaging azimuthally 360° of 2D SAXS patterns via the Fit2D software. The periodicity of the phase-separated domains (R_d) and the Guinier radius (R_g) were calculated by Equation (S5) and (S6), where q_{max} is the scattering vector at which I_q^2 shows a maximum, R_{shpere} is the spheres of monodispersed distribution.

$$R_d = 2\pi/q_{max} \tag{S5}$$

$$R_g = \sqrt{3/5}\, R_{sphere} = \sqrt{3}/q_{max} \tag{S6}$$

1.6.5. Wide-angle X-ray diffraction (WAXD) measurements

WAXD measurements were conducted on an extensometer (KL-2000N, Shanghai Qingkai Experimental Equipment Co., Ltd., China) using the Shanghai Synchrotron Radiation Facility (SSRF) (wavelength λ =0.124 nm). Each WAXD pattern obtained in the center of the sample was collected within 60 s and the strain was controlled by the extensometer at 200 μm/s. CeO_2 standard was used to calibrate the sample-to-detector distance and the value was 222.489 mm. All the WAXD patterns were background

corrected using the standard procedure. The one-dimensional WAXD curves were integrated within 360° of 2D WAXD patterns along the vertical direction via the Fit2D software.

1.6.6. Simulation of H-bonding and π-π interactions

Molecular dynamics (MD) simulation was carried out with the Materials Studio 8.0 (Accelrys. Inc.). In the MD simulations and model optimization, all force fields were based on the COMPASS force field. All-atom MD simulation systems were comprised of five polymer chains, each of which consisted of four hard and six soft segments, and the number of repeating units in the soft segments were 13. The initial configurations of simulation systems were constructed by randomly distributing the polymer chains in simulation cells with the Amorphous Cell module based on the packing technique of Theodorou and Suter[2] and Meirovitch scanning method.[3] The polymer structures were subjected to a 10-circle thermal annealing from 300 to 800 K and then back to 300 K with 50 K intervals. At each temperature, 100 ps of NPT MD simulation was performed at the constant pressure (1 bar) with a time step of 1 fs. After the 10-circle annealing, the 1 fs for NVT MD. The construction models for calculating the number of H-bonding and total cohesive energy are displayed in Figure S4, Supporting Information.

The forms of H-bonding in different samples are shown in Table S2, Supporting Information, and the criterion for the H-bonding is that the length is smaller than 2.5 Å and the angle is greater than 120 degrees. The calculated numbers of H-bond for different samples are listed in Table S2, Supporting Information.

The cohesive energy per chain is defined as the average energy per chain required to separate all the polymer chains in a condensed state into infinite distance from each other[1], which was determined according to the equation (S7):

$$E_{cohesive} = \frac{\sum_{i=1}^{5} E_{pot}^{isolated}(i) - E_{pot}^{5}}{5} \quad \text{(S7)}$$

where $E_{pot}^{isolated}$ and E_{pot}^{5} respectively represents the average potential energy of an isolated polymer chain in vacuum and a condensed system consisting of five polymer chains, whereas the potential energies of the isolated polymer chains were calculated by averaging 20 frames in 100 ps after the equilibrium simulation of 1 ns.[1] The potential energy in the condensed system was calculated by the same method after the annealing. The cohesive energy of PU(P-N), PU(P-Van-N), PU(Reduced) and PU(Van-N) were calculated and shown in Table S3, Supporting Information.

The contribution of π-π interactions between the aromatic rings and imines to the cohesive energy was calculated by removing the irrelevant groups in the simulated system. The remaining model is consisted of the corresponding unit shown in Figure S5, Supporting Information. The cohesive energy contributed from the π-π interactions is also calculated by using the equation S7, with the results shown in Table S3, Supporting Information.

1.6.7. Simulation of tensile properties

To gain a better understanding of tensile mechanism, we built the amorphous cells of PU(P-N), PU(P-Van-N), PU(Reduced) and PU(Van-N) with 3D periodic boundary condition and simulated the tensile process by virtue of MD simulation. The parameters of the models are shown in Table S4, Supporting Information.

In a simulation, the geometry optimization was first performed for each amorphous cell by using Smart Minimized method with energy convergence tolerance of 2×10^{-5} kcal/mol. This geometrically optimized amorphous cell was then further annealed from 300 to 500 K for 10 cycles with 5 heating ramps per circle. Afterward, the annealed cell was relaxed with 1000 ps under NVT (constant number of particles, volume, and temperature) ensemble at 298 K. Finally, the cell was subjected to 1000 ps of NPT (constant number of particles, pressure, and temperature) at one bar and 298 K to obtain a thermodynamic equilibrium state. The resulting cell was used to perform the tensile process in the X direction as follows: Firstly, the cell was subjected to an initial stress to produce a strain. Then, the deformed cell was equilibrated with 106 steps under NPT ensemble. Finally, the equilibrium stress-strain was obtained. All modeling and simulations were performed using Materials Studio software with the ab into condensed-phase optimized molecular potentials for atomistic simulation studies (COMPASS) force field. The pressure were controlled by Andersen barostat[4] and the temperature were controlled by Berendsen thermostat.[5]

The Newtonian equation of motion was solved by Verlet velocity time integration method with a time step of 1 fs.

The covalent interactions were calculated mainly based on interactions of bond stretch, angle bend, torsion, out-of-plane and stretch-stretch via formulas S8-S11.

$$E = \frac{K_0}{2}(R - R_0)^2 \quad \text{(S8)}$$

where E represents the interactions of bond stretch and angle bend, k_0 is the force constant, R is the bond distance and R_0 is the equilibrium bond distance.

$$E = \frac{1}{2} \sum_j B_j \left(1 - d_j \cos\left[n_j \emptyset\right]\right) \tag{S9}$$

where E represents the interaction of torsion, B_j is the barrier height, d_j and n_j is respectively the phase factor and periodicity, $\emptyset$ represents the angle between the *ijk* and *jkl* planes.

$$E = \frac{K_0}{2} \omega_{av}^2 \tag{S10}$$

where E represents the interaction of out-of-plane, K_0 is the force constant and ω_{av} is the average of the three umbrella inversions about *i*.

$$E = K_0 \left(R_{ij} - R_{ij0}\right)\left(R_{jk} - R_{jk0}\right) \tag{S11}$$

where E represents the interaction of stretch-stretch, K_0 is the force constant, R_{ij} and R_{jk} is bond distance from bond stretch, and R_{ij0} and R_{jk0} is equilibrium bond distance from bond stretch.

The van der Waals interactions were computed through the Lennard-Jones function with a cutoff distance of 12.5 Å via formula S12, and the electrostatic interactions were calculated by the Ewald method with an accuracy of 0.001 kcal/mol via formula S13.[5]

$$E = D_0 \left[2\left(\frac{R_0}{R}\right)^9 - 3\left(\frac{R_0}{R}\right)^6\right] \tag{S12}$$

where D_0 is the equilibrium well depth, R is the distance, and R_0 is the equilibrium distance.

$$E = C \frac{q_i q_j}{\varepsilon R} \tag{S13}$$

where C is a unit conversion factor, q_i and q_j are the charges on atoms *i* and *j* respectively, *R* is distance, and ε is the relative dielectric constant.

1.6.8. Syntheses of the Si/C anode

Firstly, Si/C powder (450 mAh g^{-1}), acetylene black conductive carbon, and PU(P-Van-N) binder with a mass ratio of 8:1:1 were mixed in *N*-methylpyrrolidone (NMP) to form a homogeneous slurry. Then the slurry was coated onto the current collector *via* a common doctor-blade coating method, with the active material loading to about 3 mg cm^{-2}. Finally, the Si/C anode was prepared by heating at 100 °C for 12 hours.

1.6.9. Assembly of half-cells

Firstly, the working electrodes without calendaring were fabricated by cutting out round disks with a 12 mm diameter and further dried overnight to remove moisture. Then the coin-type cells were assembled in a glove box filled with pure Ar, and a Li foil and a polypropylene micro-porous film (Celgard 2400) were used as the counter electrode and the separator, respectively. EC/DEC/FEC-based (1:1:0.1 by volume) electrolyte containing 1 M LiPF6 was used.

1.6.10. Electrochemical test

To evaluate electrochemical performance, a galvanostatic charge-discharge cycling test was carried out using LAND battery cycler (China) between 0.01 and 1.5 V. The cells were cycled at a rate of C/25 for first cycle and then C/5 for additional cycles. All the tests were performed at room temperature (25~30°C).

1.6.11. Fabrication of AgNW/PU electrodes

Ag nanowires (AgNWs) were synthesized by a modified method.[6] The fabrication steps of the composite electrode are illustrated in Figure S34a, Supporting Information.

Firstly, a dispersion of AgNWs in deionized water (~2 mg ml^{-1}) was vacuumized to deposit onto the filter membrane through a mask. Then the AgNW/filter membrane was attached onto the surface of PU film (50 mm × 25 mm), which was cleaned gently with ethanol and placed on a flat surface. The AgNW film was inlaid on the surface of PU by the UV/ozone irradiation treatment (Sen Lights Corporation, Japan) for 20 min. Then, the filter membrane was wetted by water and peeled off from the PU film, and the conductive AgNW/PU film was obtained. For stretchability and bending tests, silver paste was printed on both ends of the resulting AgNW/PU films and dried for at room temperature. For fire test, a strip of AgNW was directly coated onto the surface of PU film, which is shown in Figure S35a, Supporting Information.

1.6.12. Characterization of AgNW/PU

The real-time resistances of the AgNW/PU devices were measured by connecting the two ends of the composite film to a digital LCR meter (34465A, Keysight Technologies). A tensile testing machine (Home-made Linear stage) was applied to stretch the electrodes at speeds of 0.5 mms. Continuous stretching-relaxation tests over 500 and 100 cycles were carried out with peak strains of 10% and 50%, respectively. A bending testing machine (Home-made Linear stage) was applied to bend and relax the electrodes from 20 to 10 mm over 200 cycles at speeds of 0.5 mms. The change in the resistance of the electrodes can be expressed as (ΔR=(R-R_0))/R_0, where R_0 is the initially measured resistance and R is the value measured after substrate stretching or bending. The morphology of the AgNWs was observed by using a scanning probe microscope (FEI Nova NanoSEM 450) and TEM (Phillips CM 200).

2. Supplementary Figures and Tables

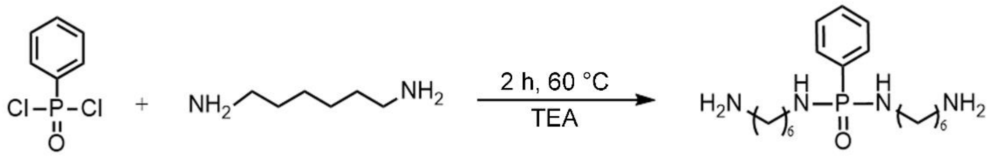

Figure S1. The synthetic routes for P-N.

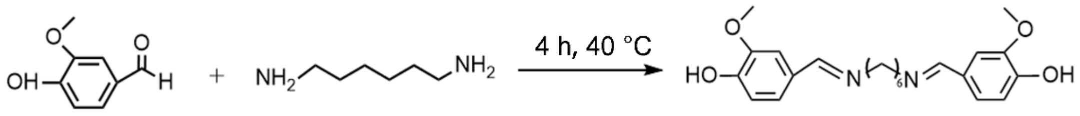

Figure S2. The synthetic routes for Van-N.

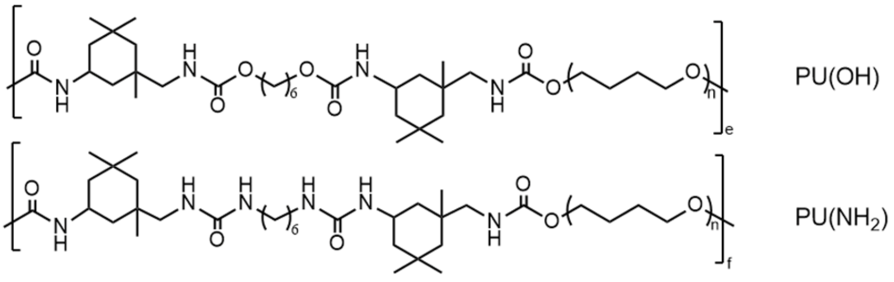

Figure S3. Chemical structure for PU(OH) and PU(NH_2).

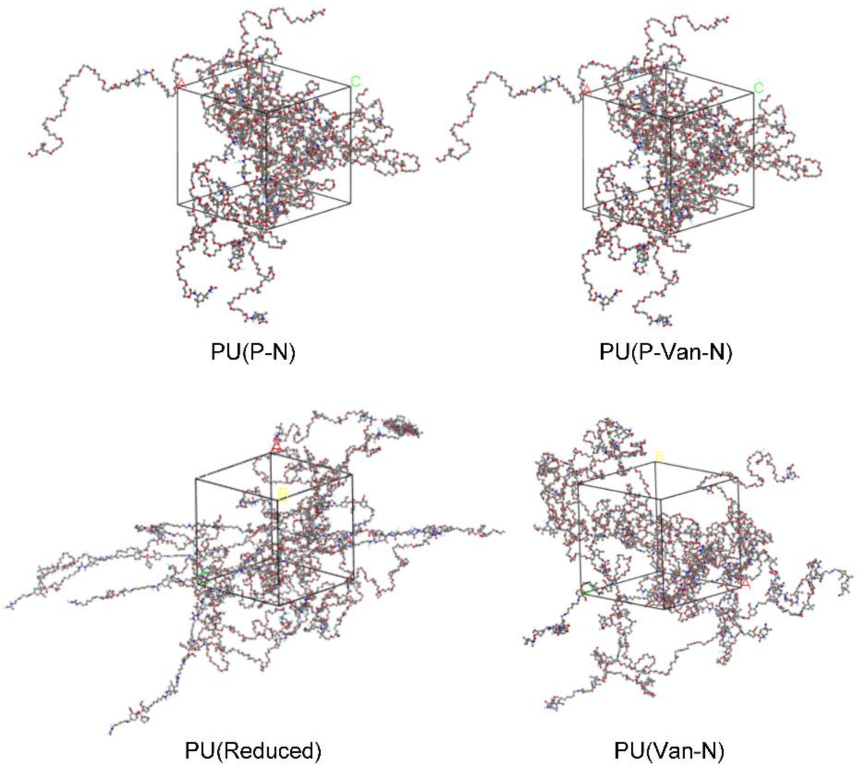

Figure S4. The construction model for calculating the number of H-bonding and total cohesive energy.

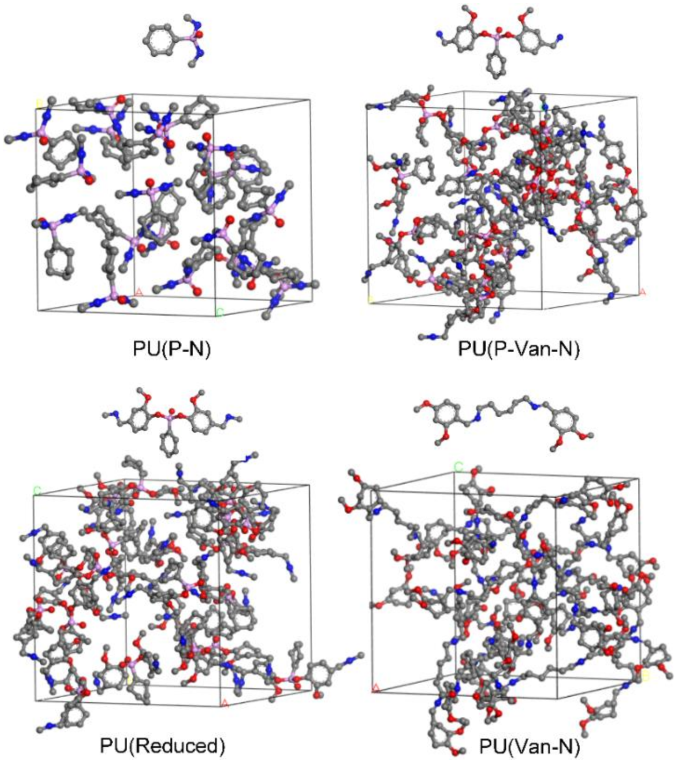

Figure S5. The unit and the corresponding models for calculating the cohesive energy of π-π interactions.

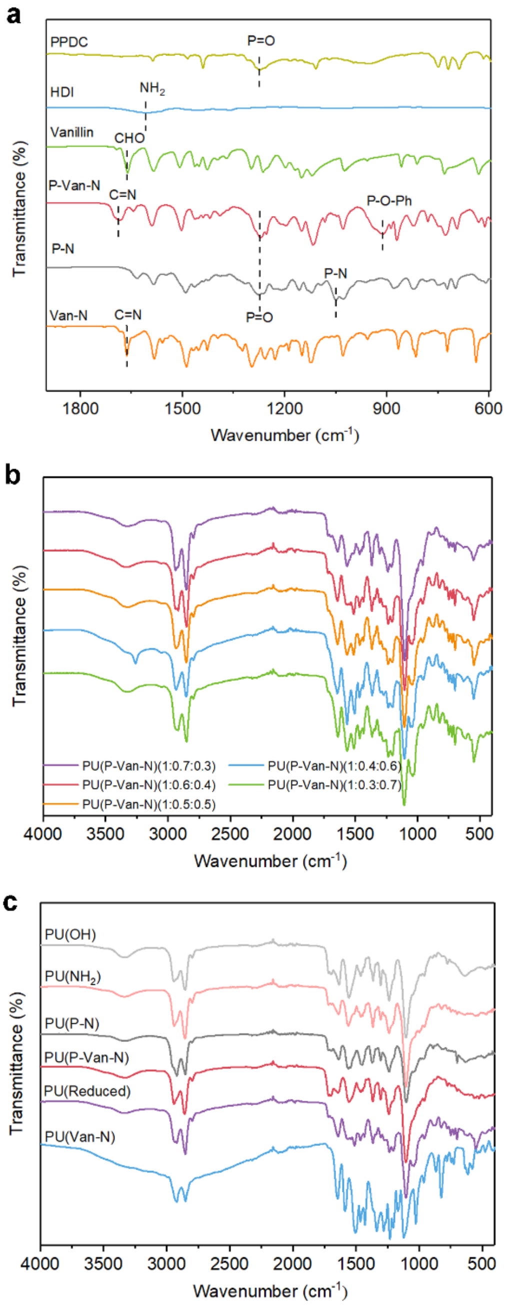

Figure S6. a) FTIR spectra of P-Van-N, P-N and Van-N. FTIR spectra of PUs b) with different ratio of P-Van-N and PTMEG and c) with different hard segments.

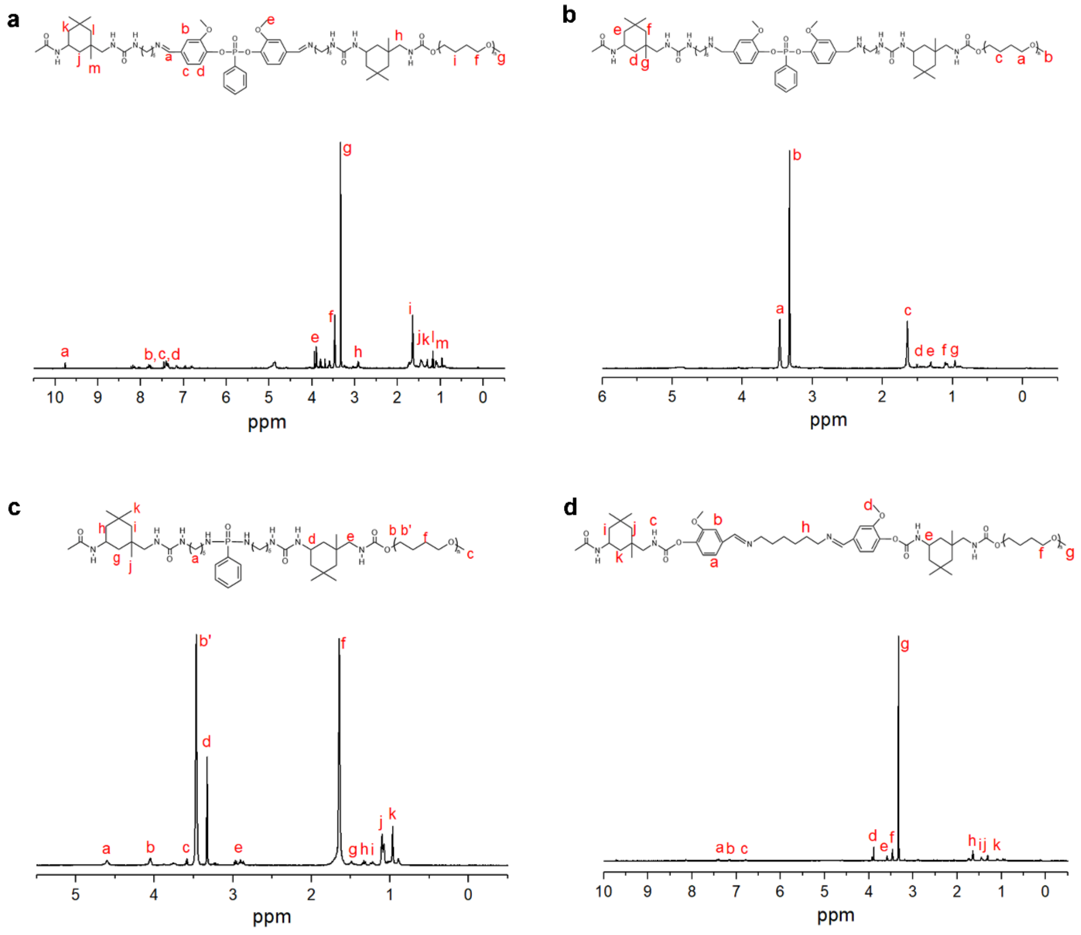

Figure S7. ^{1}H NMR (500 MHz, $CDCl_3$) spectrum of the a) PU(P-Van-N), b) PU(Reduced), c) P-N and d) PU(Van-N).

Table S1. GPC results for PUs.

Samples	M_w g mol^{-1}	PDI (M_w/M_n)
PU(P-Van-N)(1:0.7:0.3)	22,700	2.016
PU(P-Van-N)(1:0.6:0.4)	65,700	2.085
PU(P-Van-N)(1:0.5:0.5)	24,400	1.719
PU(P-Van-N)(1:0.4:0.6)	45,900	1.717
PU(P-Van-N)(1:0.3:0.7)	26, 500	2.138
PU(OH)	15,600	2.170
PU(NH_2)	21,500	1.673
PU(P-N)	31,500	2.066
PU(Reduced)	55,300	2.076
PU(Van-N)	42,500	1.309

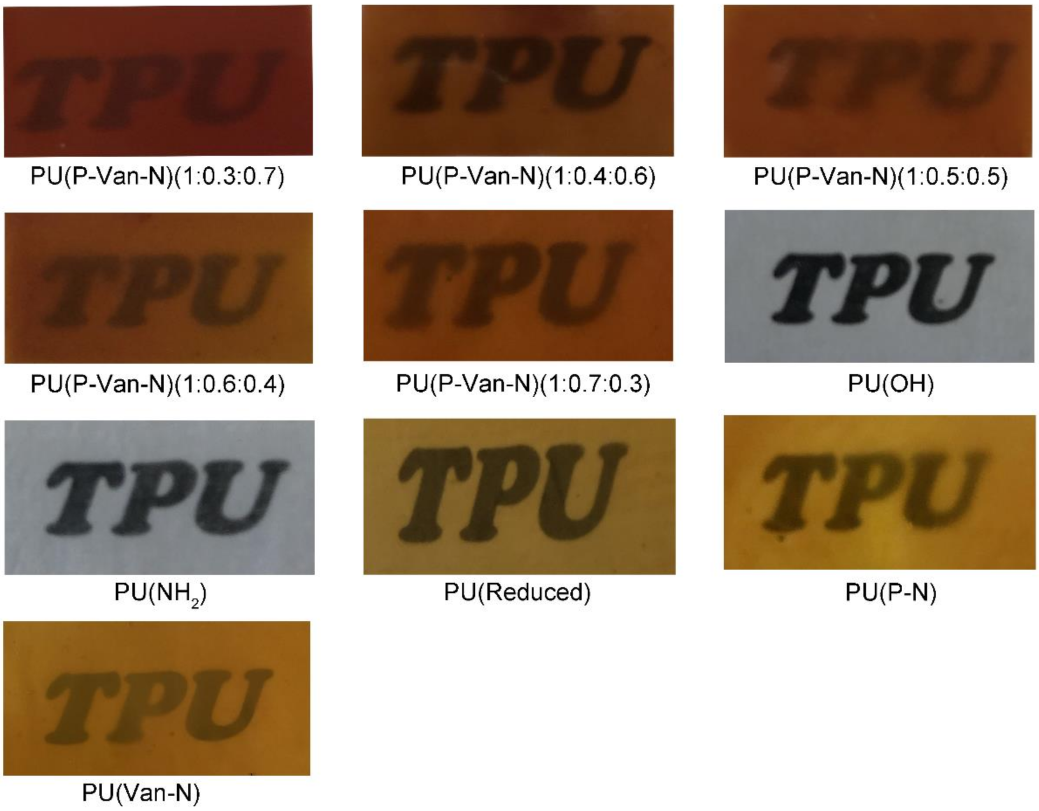

Figure S8. The digital pictures of prepared PU films.

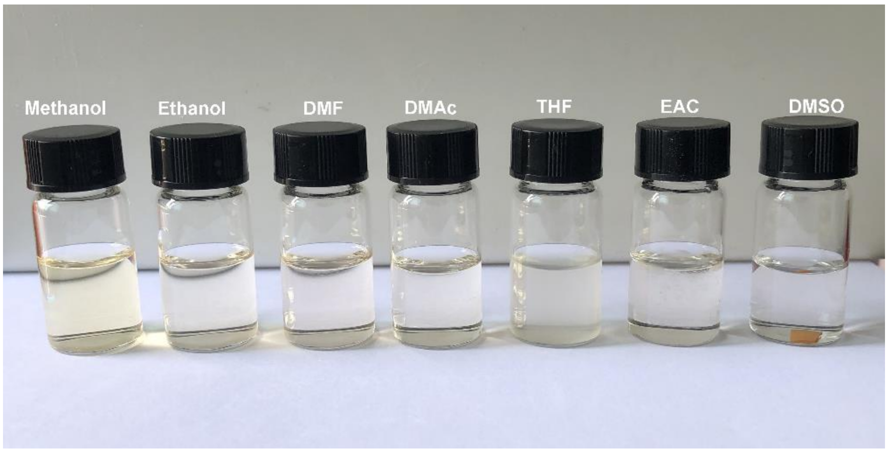

Figure S9. The solubility of PU(P-Van-N) in common solvents.

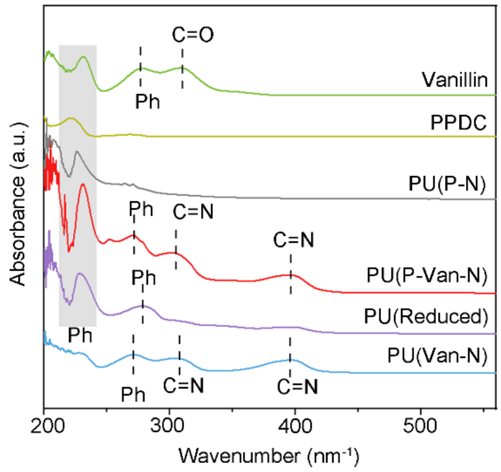

Figure S10. UV-Vis spectra for PU(P-Van-N), PU(Reduced), PU(P-N) and PU(Van-N).

Table S2. The diagram and numbers of H-bond.

Samples	The diagram of H-bond	The number of H-bond
PU(P-N)	N-H···O=C	50
PU(P-Van-N)	N-H···O=C, N-H···N=C	35
PU(Reduced)	N-H···O=C	42
PU(Van-N)	N-H···O=C, N-H···N=C	16

Table S3. The results for the contribution of π-π interactions to the total cohesive energy.

Samples	Cohesive energy (kJ/mol)		Contribution of π-π interactions (%)
	Total	π-π interactions	
PU(P-N)	315.2654	37.3632	11.9
PU(P-Van-N)	402.4364	104.4742	26.6
PU(Reduced)	390.3508	58.6686	15.0
PU(Van-N)	397.0387	86.4802	21.8

Table S4. The detailed parameters of the simulation.

Samples	The number of chains	M_n (g mol^{-1})	Cell size (Å^3)	Density (g cm^{-3})
PU(P-N)	5	24468	46.098^3	1.038
PU(P-Van-N)	5	26612	47.187^3	1.055
PU(Reduced)	5	26644	47.179^3	1.053
PU(Van-N)	5	24708	46.155^3	1.045

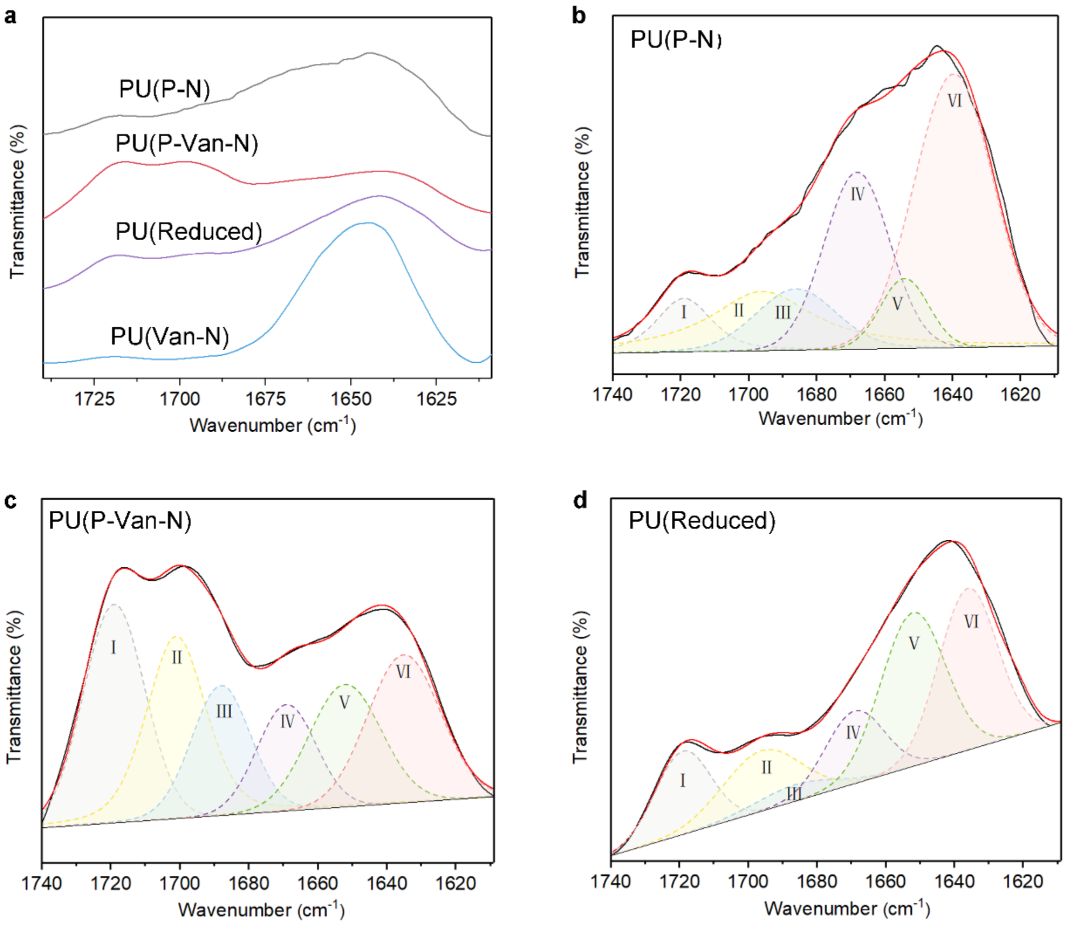

Figure S11. a) FTIR spectra of the PU(P-N), PU(P-Van-N), PU(Reduced) and PU(Van-N) in the C=O stretching region. Schematic diagram of the deconvoluted subpeaks in the FTIR C=O absorption bands for the b) PU(P-N), c) PU(P-Van-N) and d) PU(Reduced).

Table S5. Summary of the assignment of the deconvoluted subpeaks in the FTIR C=O absorption bands for the PU(P-N), PU(P-Van-N) and PU(Reduced) elastomers.

Assignment		Wavenumber (cm^{-1})			Area (%)		
		PU (P-N)	PU (P-Van-N)	PU (Reduced)	PU (P-N)	PU (P-Van-N)	PU (Reduced)
υ (C=O) urethane amide	Free	1719	1719	1718	6.6	22.6	13.2
	H-bonded (Ordered)	1696	1696	1695	15.2	20.1	13.5
υ (C=O) urea amide	Free	1686	1684	1686	9.6	13.3	3.8
	H-bonded (Disordered)	1668	1669	1668	21.8	10.7	12.1
	H-bonded (Disordered)	1654	1652	1651	6.3	15.1	28.2
	H-bonded (Ordered)	1639	1636	1636	40.4	18.1	26.7
**Total degree of H-bonded**					83.7	74.0	80.5

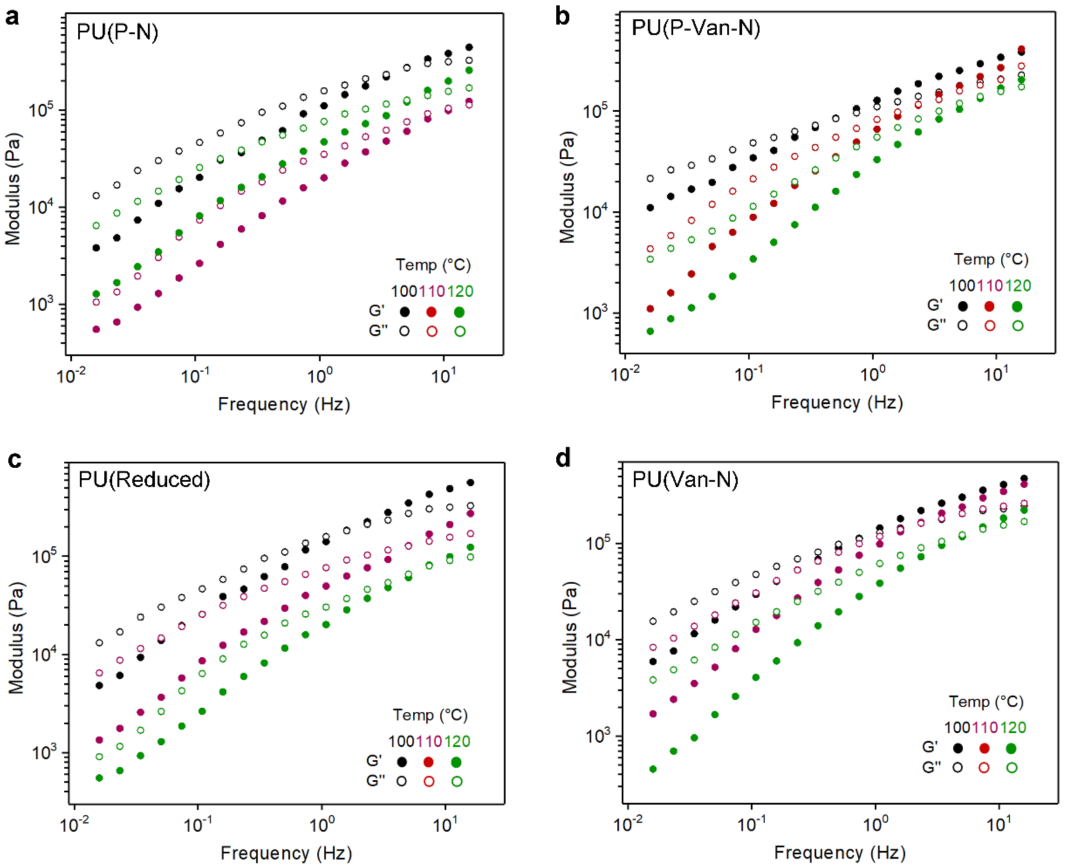

Figure S12. G' and G'' values of a) PU(P-N), b) PU(P-Van-N), c) PU(Reduced) and d) PU(Van-N) recorded in frequency (ω) dispersion tests.

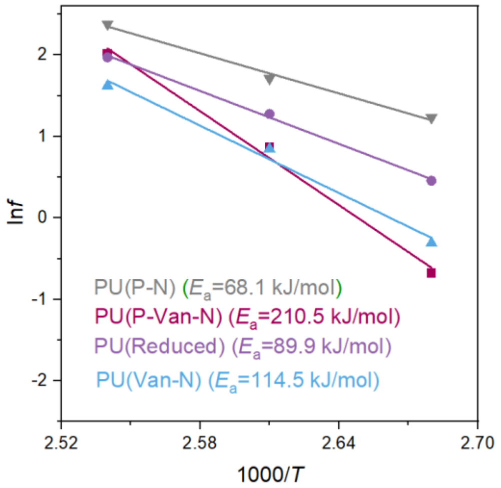

Figure S13. Arrhenius plots of the intersection frequencies ($f = \omega/2\pi$) for the G' and G'' curves of PU(P-N), PU(P-Van-N), PU(Reduced) and PU(Van-N) estimated from frequency sweep tests.

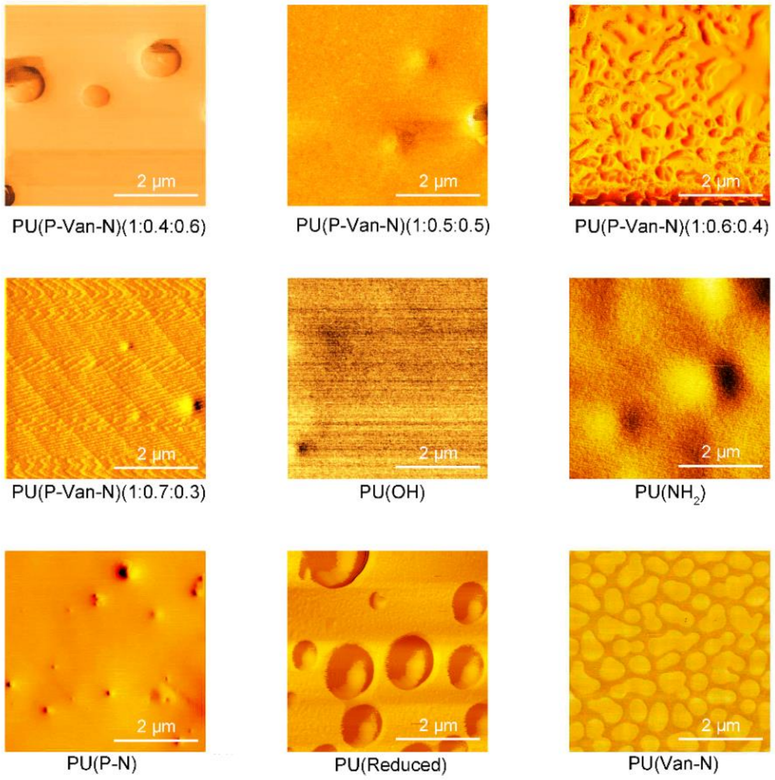

Figure S14. AFM phase images for PUs.

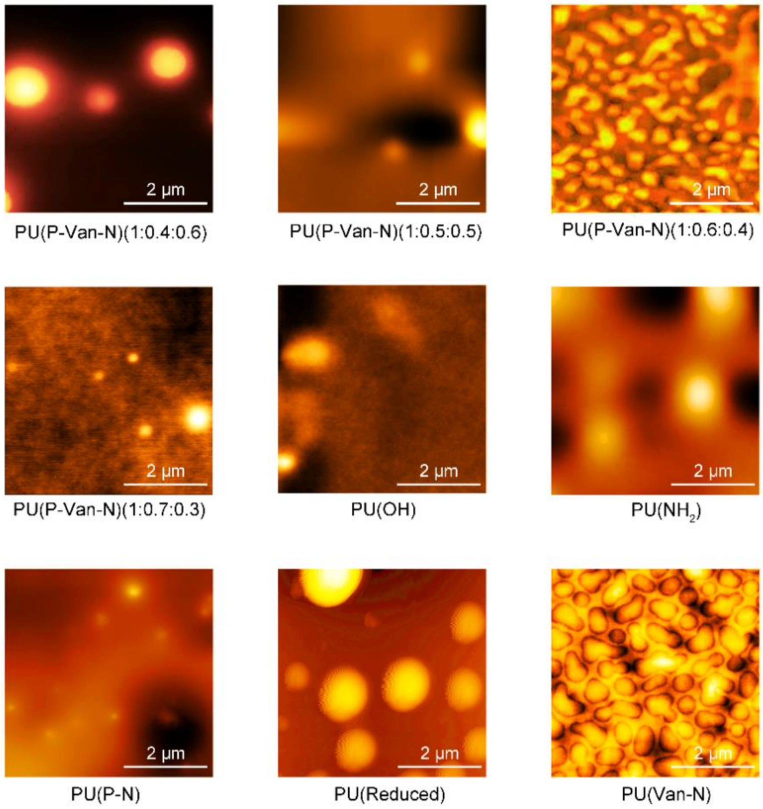

Figure S15. AFM height images for PUs.

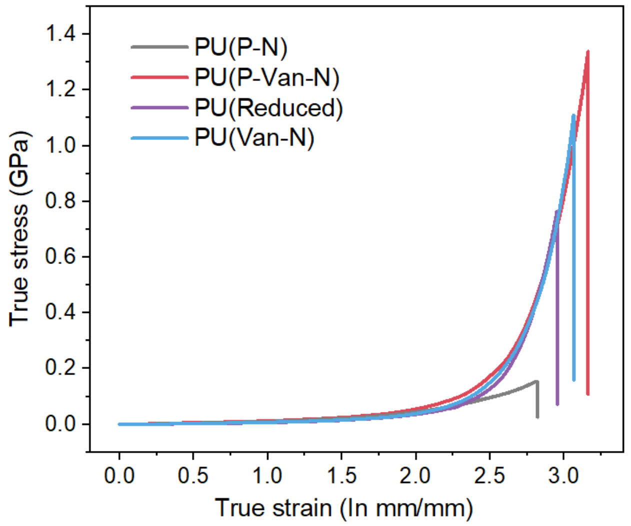

Figure S16. True stress-strain curves of the PUs.

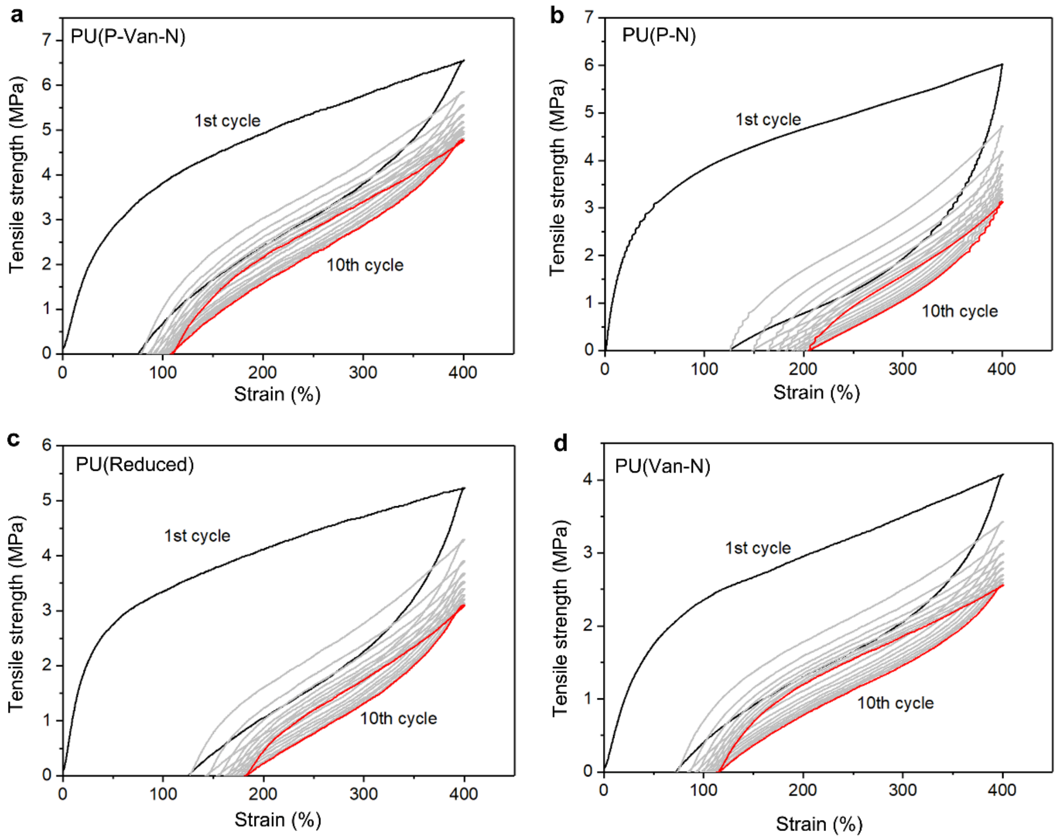

Figure S17. Cyclic tensile test curves in successive loading-unloading cycles at 400% strain for a) PU(P-Van-N), b) PU(P-N), c) PU(Reduced) and d) PU(Van-N).

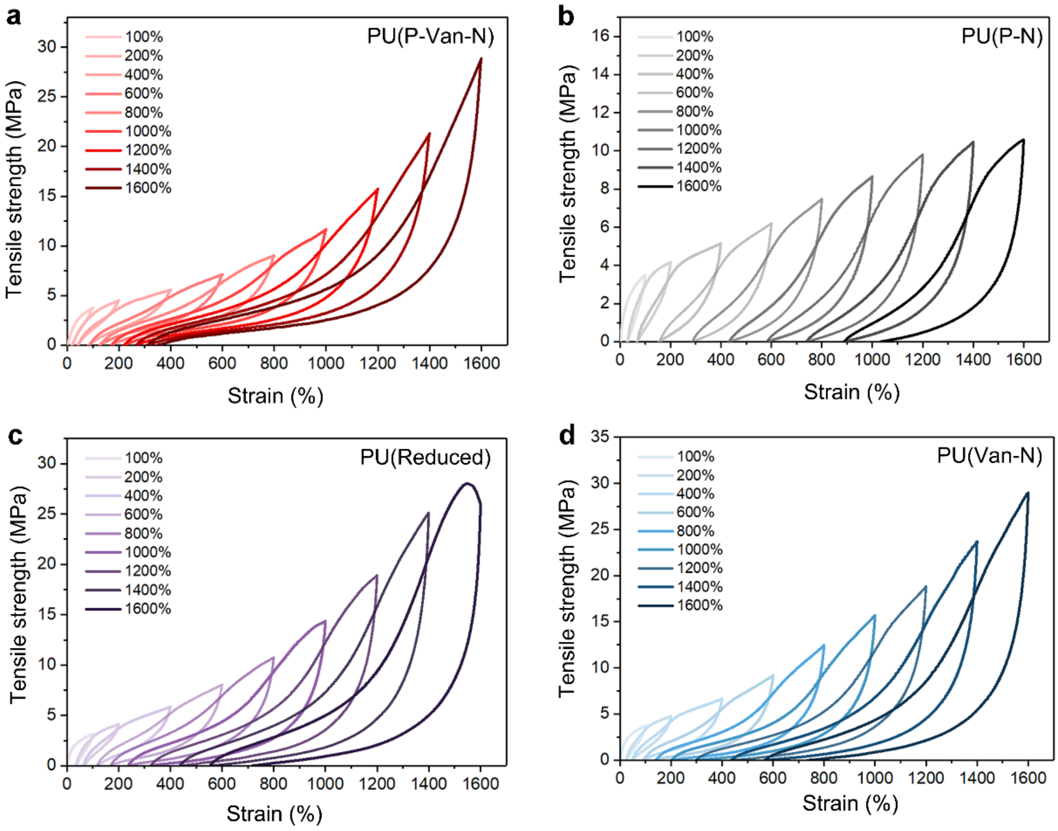

Figure S18. Cyclic tensile test curves in successive loading-unloading cycles with increasing strains for a) PU(P-Van-N), b) PU(P-N), c) PU(Reduced) and d) PU(Van-N).

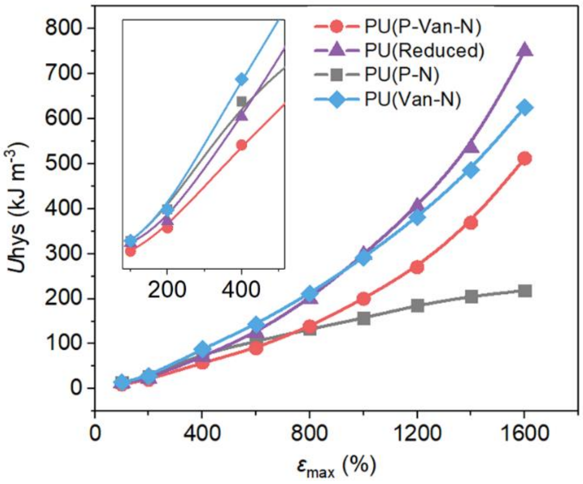

Figure S19. The hysteresis area at each loading-unloading cycle for PU(P-Van-N), PU(P-N), PU(Reduced) and PU(Van-N).

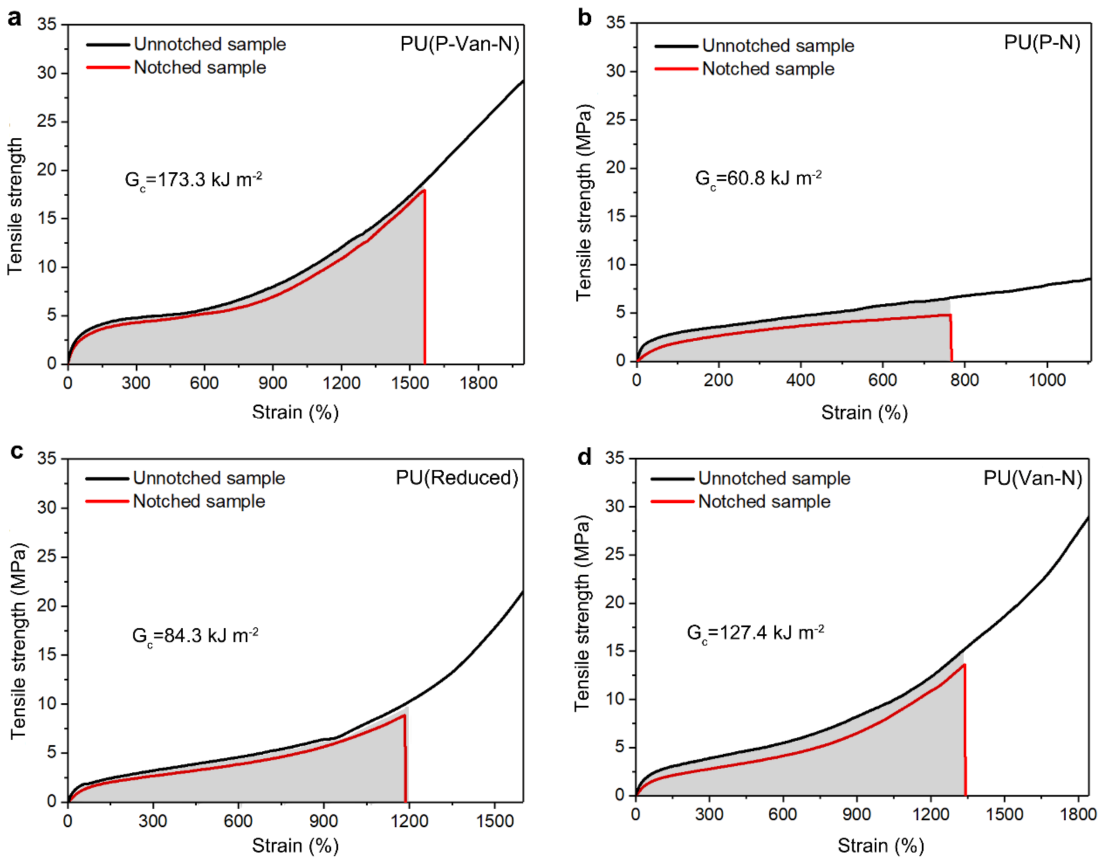

Figure S20. Typical stress-strain curves of the unnotched and notched sample for a) PU(P-Van-N), b) PU(P-N), c) PU(Reduced) and d) PU(Van-N).

Table S6. The performance comparison for the PU(P-Van-N) elastomer and some of the literature-reported PU elastomers.

Samples	Tensile strength (MPa)	Elongation at break (%)	Toughness (MJ m^{-3})	Fracture energy (kJ m^{-2})	Fire-retarding (Yes/No)	Reference in SI/main manuscript
Our work	56	2200	460.5	173.3	Yes	-
DA-PU	25	1900	175.9	-	No	[7]/[2]
PR-PDMS-2	1.05	2800	19	6.8	No	[8]/[5]
P-Cur-Eu	1.8	900	~7	2.4	No	[9]/[14]
PS	45.06	1890	~450	-	No	[10]/[6]
PPG-MPU	70	500	270	-	No	[11]/[3]
PDMS-MPU0.4-IU0.6	1.5	1700	~14	12.0	No	[12]/[55]
PU-BPY0.5-Zn	52.4	~2170	363.8	192.9	No	[13]/[7]
PT-HM-U20	48.5	2580	386.5	-	No	[14]/[27]
PDMS-MPI-HDI	0.26	1200	~2	-	No	[15]/[4]
PDMS(30)-g-BuP	4.3	350	~15	-	No	[16]/[35]
IPDI-SPU2000	75.6	1520	390.2	215.2	No	[1]/[26]
PU-UPy-DOPA-Fe	~47	~2000	~405	-	No	[17]/[1]
PUI-3	40.7	836	~192	-	No	[18]/[39]
COVPU 5	~21	~230	~31	-	No	[19]/[13]
ASB-WPU-2	14.3	942.6	64.8	-	No	[20]/[54]
POUs	~52	~700	~154	-	No	[21]/[19]
0.5%-AD/GO-PU	78.3	1273.2	505.7		No	[22]/[33]
1.35%PEPE-TPU	39.2	1471	~280	-	Yes	[23]/[16]
SPUs	24	348	~45	-	Yes	[24]/[11]
TPIU/12.4%PDMS	20.1	747	~75	-	Yes	[25]/[15]
boron-containing PU	3.06	178	~3	-	Yes	[26]/[12]
TPU/PPBPP12	16.6	812.1	~75	-	Yes	[27]/[10]

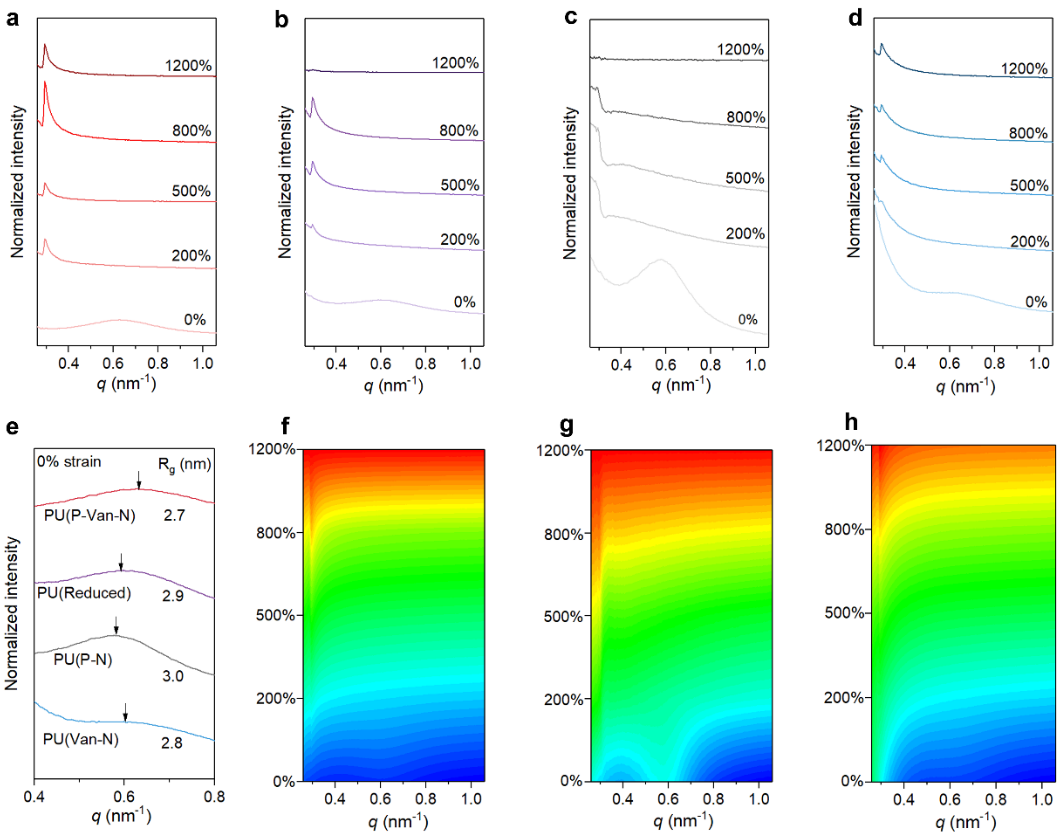

Figure S21. 1D integrated SAXS curves for a) PU(P-Van-N), b) PU(Reduced), c) PU(P-N) and d) PU(Van-N) with increasing strain. e) 1D integrated SAXS curves at 0% strain. The corresponding contour maps for f) PU(Reduced), g) PU(P-N) and h) PU(Van-N).

Table S7. The parameters calculated from 1D SAXS curves of PU(P-Van-N).

Strain (%)	R_d (nm)	R_g (nm)
0	10.0	2.7
200-1200	21.7	6.0

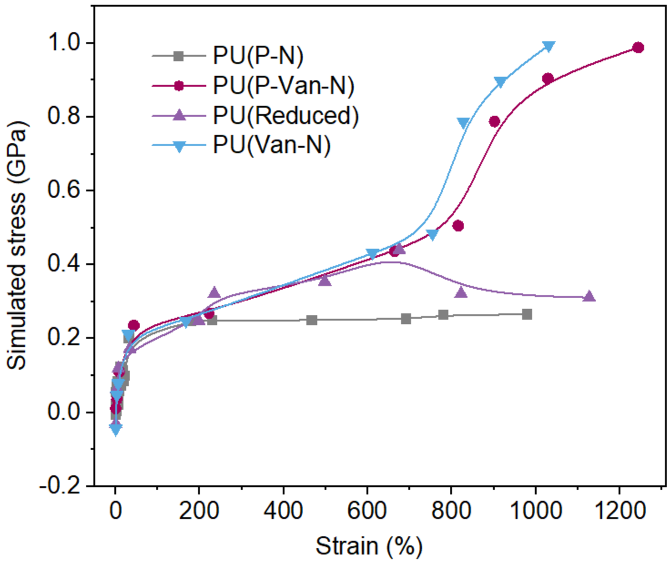

Figure S22. Stress-strain curves of PUs based on MD simulation.

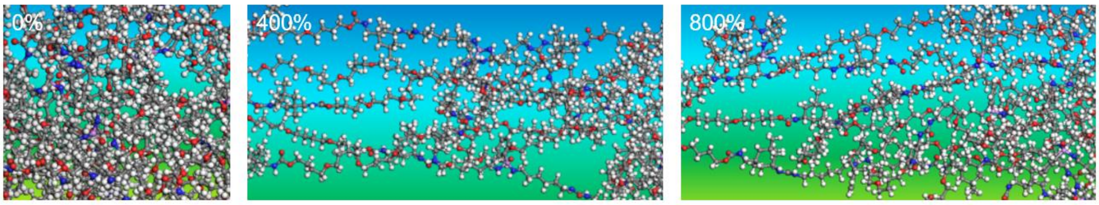

Figure S23. Snapshots showing the MD simulations of the PU(P-N) at strains of 0%, 400%, 800%.

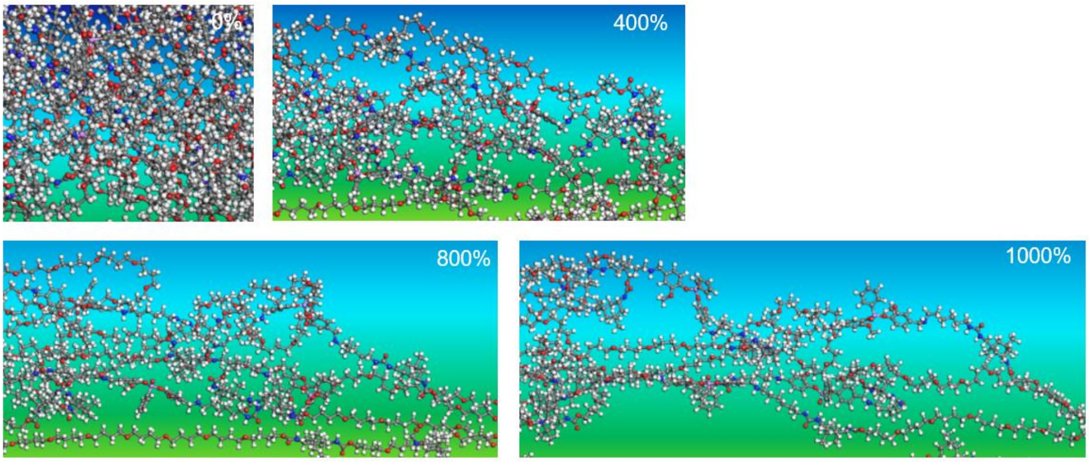

Figure S24. Snapshots showing the MD simulations of the PU(Reduced) at strains of 0%, 400%, 800%, 1000%.

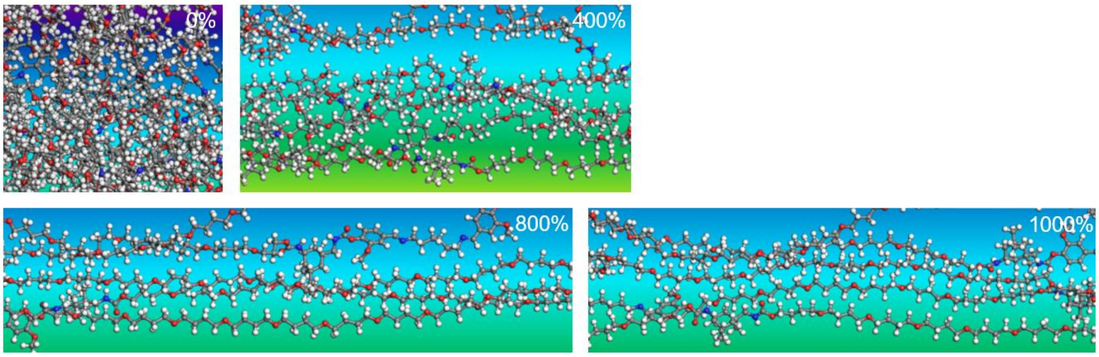

Figure S25. Snapshots showing the MD simulations of the PU(Van-N) at strains of 0%, 400%, 800%, 1000%.

Table S8. The flame retardancy for PU elastomers in LOI, UL-94 and MCC tests.

Sample	LOI (%)	UL-94 rating	TPHRR (°C)	THR (kJ/g)	PHRR (W/g)	HRC (J/g·K)
PU(P-Van-N) (1:0.7:0.3)	31.5	V-0	-	-	-	-
PU(P-Van-N) (1:0.6:0.4)	32.8	V-0	431.4	31.0	400.6	393
PU(P-Van-N) (1:0.5:0.5)	33.0	V-0	432.1	31.2	400.5	393
PU(P-Van-N) (1:0.4:0.6)	33.1	V-0	-	-	-	-
PU(P-Van-N) (1:0.3:0.7)	33.3	V-0	-	-	-	-
PU(OH)	27.5	V-2	440.4	33.7	558.1	547
PU(NH_2)	28.5	V-2	-	-	-	-
PU(P-N)	29.0	V-0	431.4	32.7	510.6	502
PU(Reduced)	25.3	V-2	434.2	32.3	511.0	503
PU(Van-N)	29.7	V-2	437.8	30.3	434.6	426

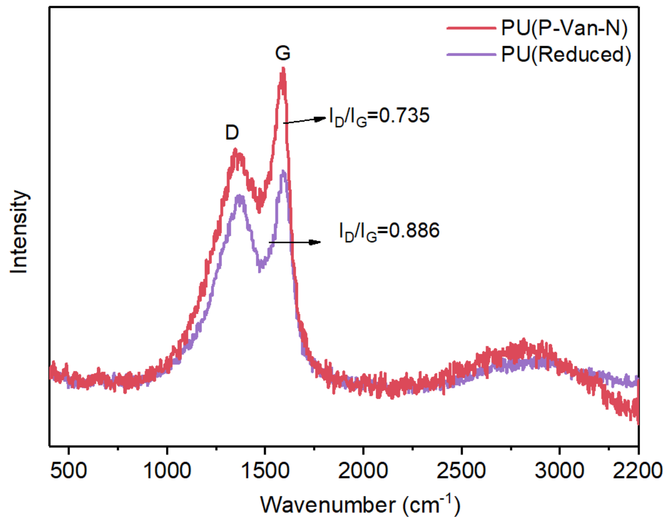

Figure S26. Raman spectra for PU(P-Van-N) and PU(Reduced).

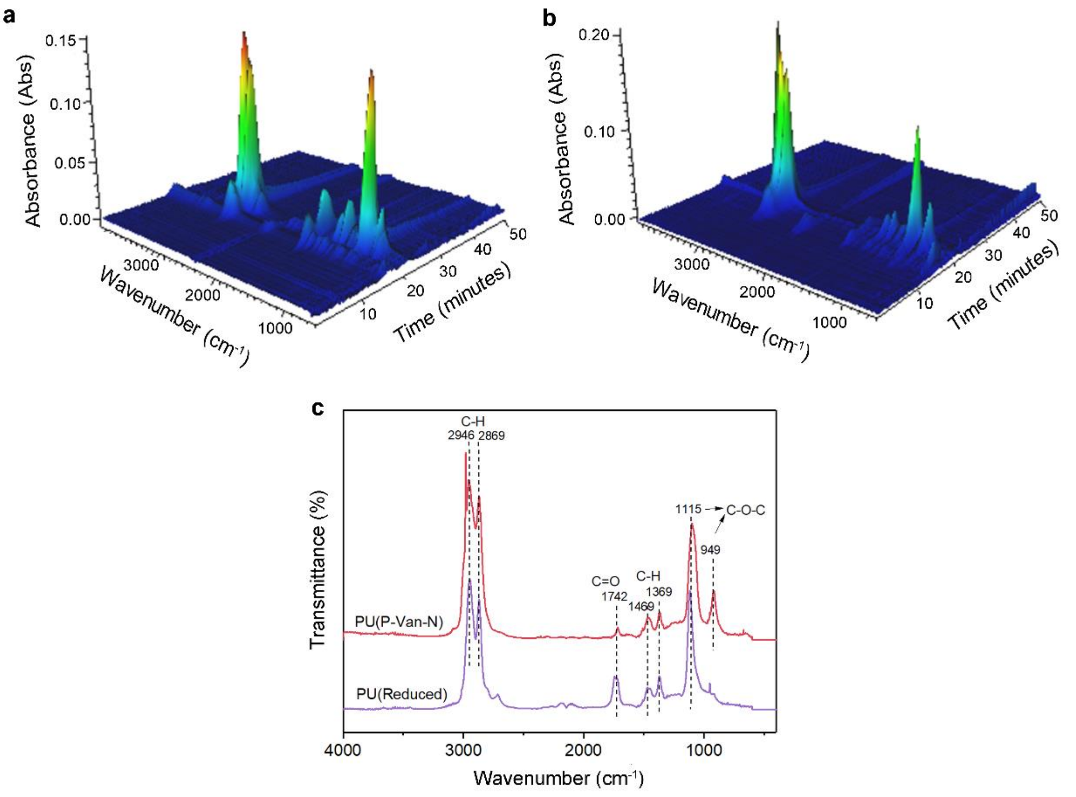

Figure S27. 3D TG-IR spectra of pyrolysis gaseous products of a) PU(Reduced) and b) PU(P-Van-N). c) FTIR absorbance spectra of pyrolysis gases from PU(Reduced) and PU(P-Van-N) at the maximum rate of degradation.

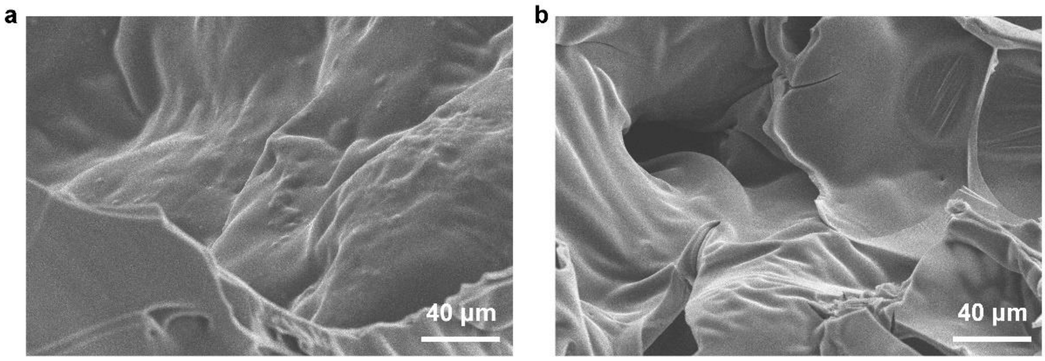

Figure S28. SEM images for char residue of a) PU(P-Van-N) and b) PU(Reduced).

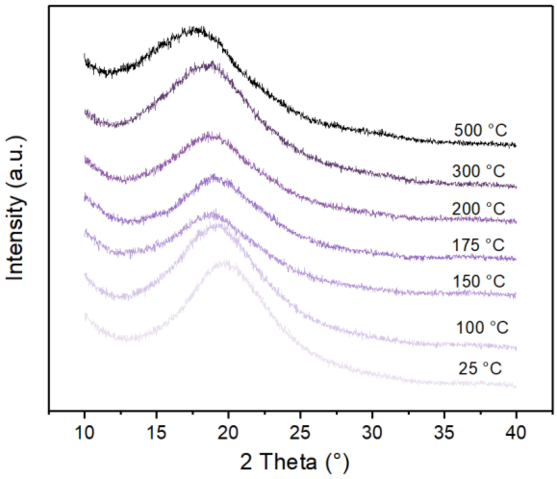

Figure S29. XRD patterns of PU(Reduced) during the heating process.

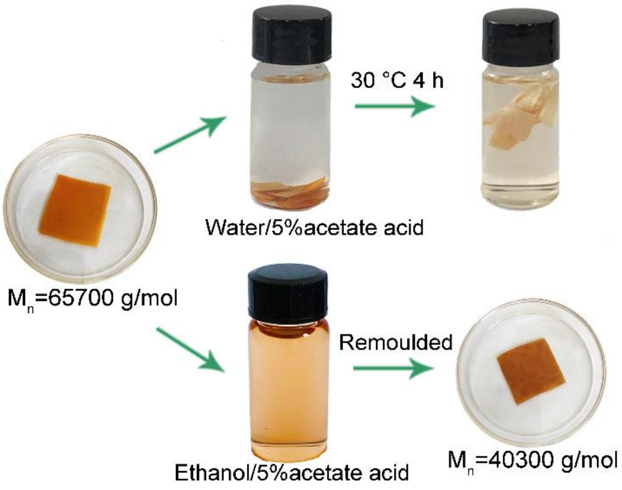

Figure S30. The degradation process of PU(P-Van-N) in different solvents.

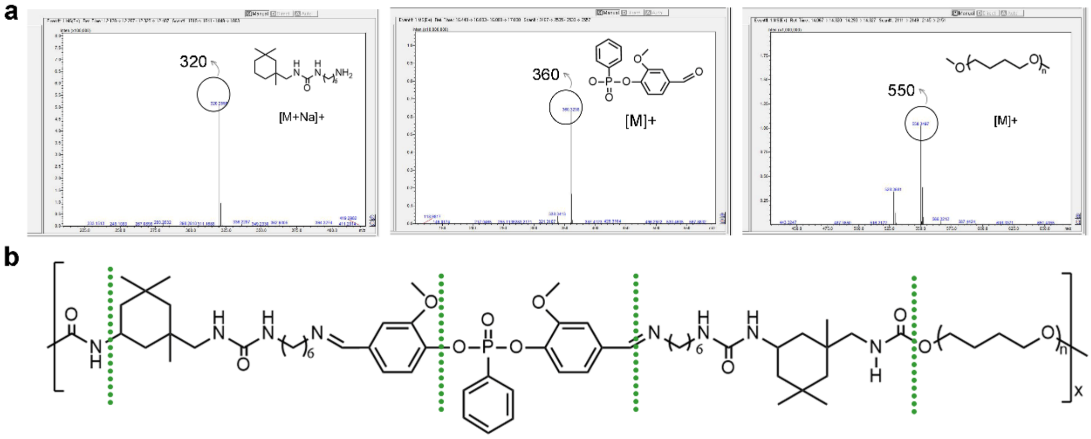

Figure S31. a) The mass spectra of the products degraded from PU(P-Van-N) and b) The diagram of degradation for PU(P-Van-N) in the ethanol/5 wt% acetic acid.

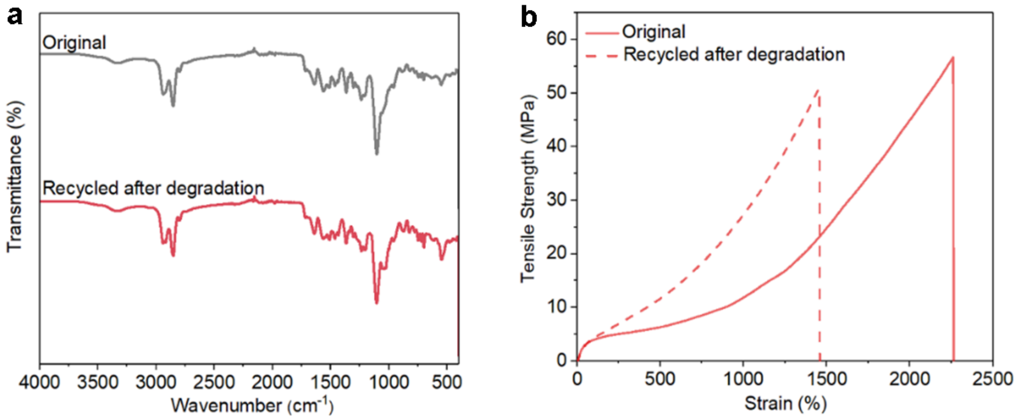

Figure S32. a) IR spectra and b) The stress-strain curves of the PU(P-Van-N) before and after degradation.

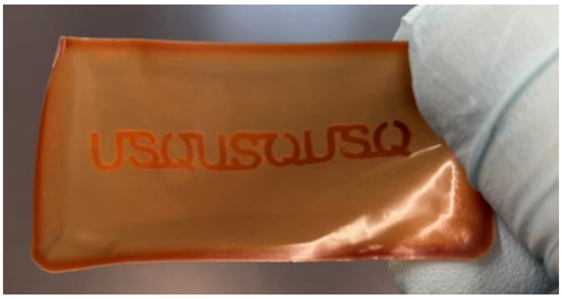

Figure S33. The digital image of as-prepared flexible AgNW/PU strain sensors.

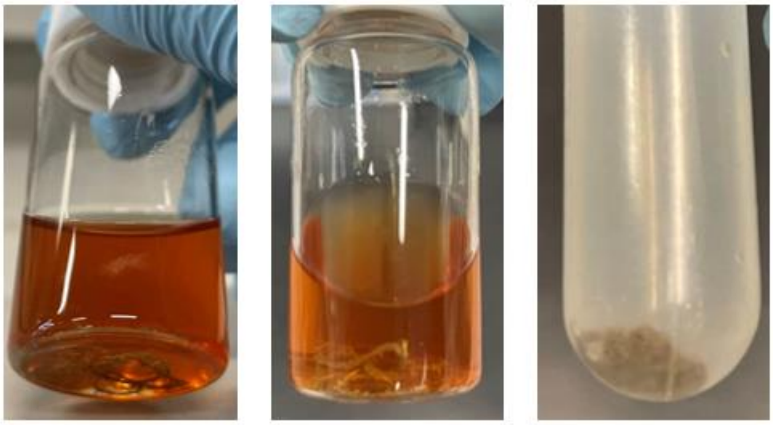

Figure S34. The recycling process of AgNW from AgNW/PU film.

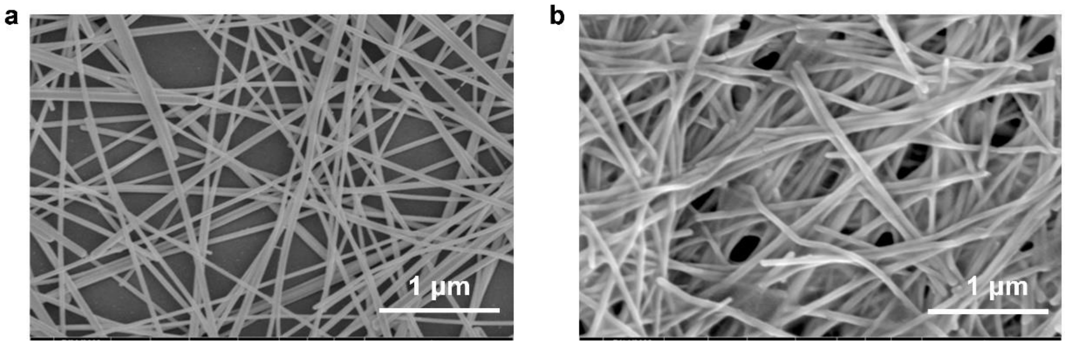

Figure S35. The morphology of the AgNW (a) freshly synthesized and (b) after recycled.

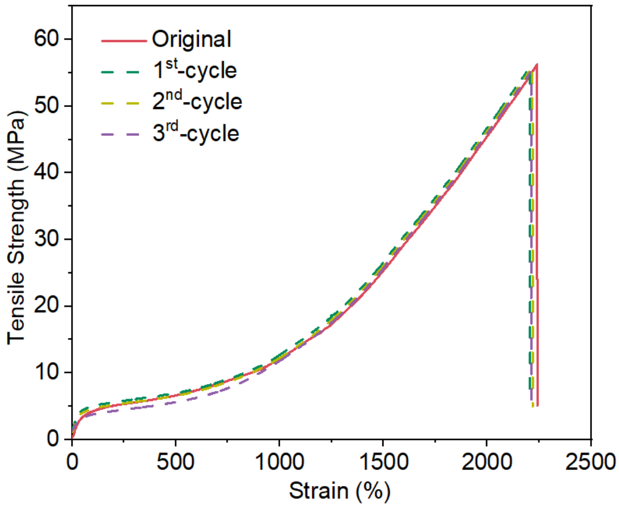

Figure S36. The mechanical properties of PU(P-Van-N) after recycling using ethanol.

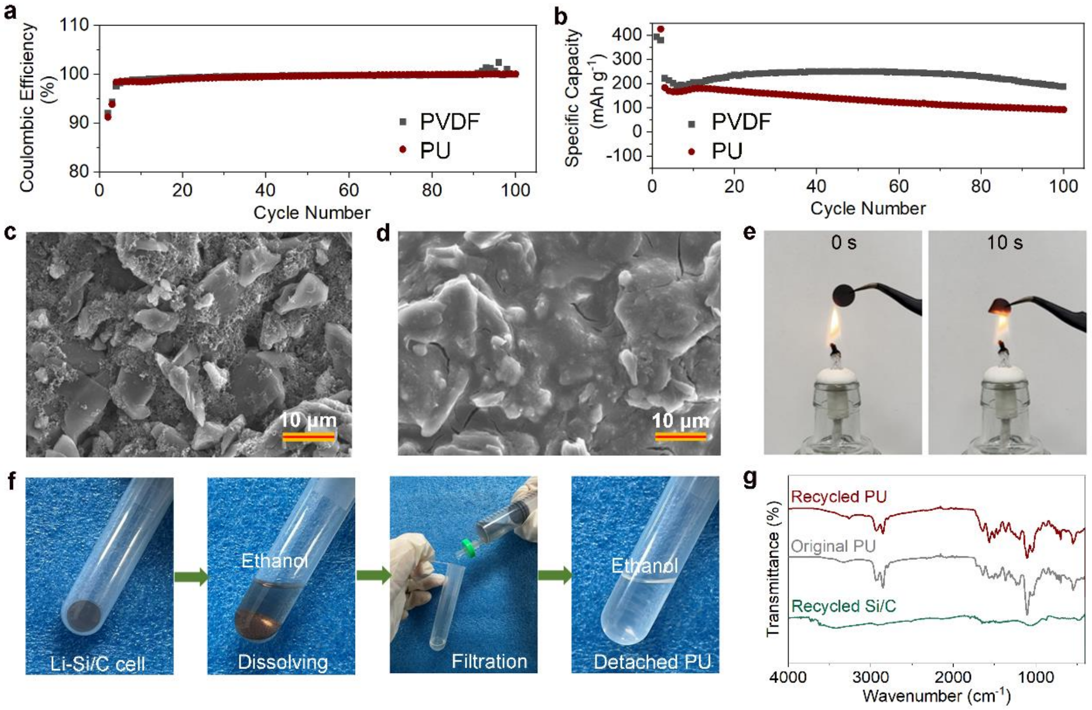

Figure S37. PU(P-Van-N) as a fire-safe and recyclable binder for Li-Si/C battery anode. a,b) Cycling performance of Li-Si/C cells based on PVDF and PU(P-Van-N) binders at 0.2 C for 100 cycles. SEM images for Li-Si/C cells based on PU(P-Van-N) binder c) before and d) after electrochemical test. e) Flame-retardant properties of the Si/C anode using the PU(P-Van-N) as the binder. f) Recycling process of PU(P-Van-N) binder from Li-Si/C cell. g) IR spectra of PU(P-Van-N) before and after recycling, and recycled Li-Si/C without PU binder.

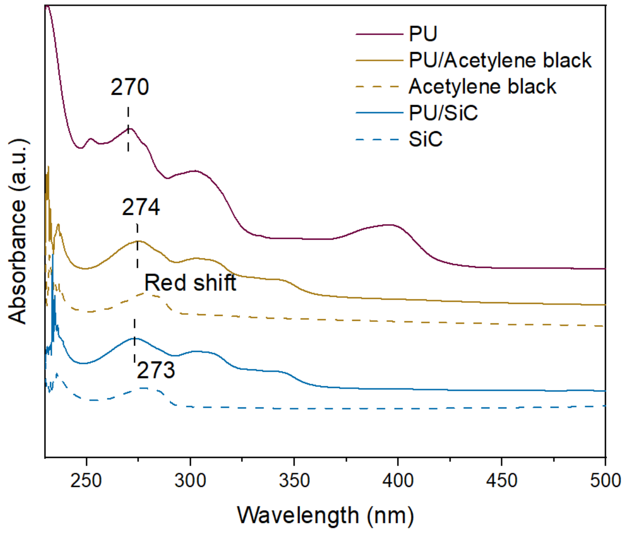

Figure S38. UV-Vis spectra for single and composite materials in Li-Si/C cell.

3. Supplementary Movies

Movie S1. The simulated stretching process of PU(P-Van-N).

Movie S2. The stretching process of AgNW/PU film.

Movie S3. The bending process of AgNW/PU film.

Movie S4. The operation of mechanical arm by coated with PU(P-Van-N) film.

Movie S5. The fire attack of AgNW/PU film.

Movie S6. Flame-retardant properties of the Si/C anode using the PU(P-Van-N) as the binder.

4. Reference

[1] Z. Q. Li, Y. L. Zhu, W. W. Niu, X. Yang, Z. Y. Jiang, Z. Y. Lu, X. K. Liu, J. Q. Sun, *Adv. Mater*. **2021**, *33*, 2101498.

[2] D. N. Theodorou, U. W. Suter, *Macromolecules* **1986**, *19*, 139-154.

[3] H. Meirovitch, *J. Chem. Phys.* **1983**, *79*, 502-508.

[4] H. C. Andersen, *J. Chem. Phys.* **1980**, *72*, 2384-2393.

[5] M. Rahmati, H. Modarress, R. J. P. Gooya, *Polymer* **2012**, *53*, 1939-1950.

[6] Y. Z. Zhu, T. Wan, P. Y. Guan, Y. T. Wang, T. Wu, Z. J. Han, G. C. Tang, D. W. Chu, *J. Colloid Interf. Sci.* **2020**, *566*, 375-382.

[7] W. B. Ying, G. Y. Wang, Z, Y. Kong, C. K. Yao, Y. B. Wang, H. Hu, F. L. Li, C. Chen, Y. Tian, J. W. Zhang, R. Y. Zhang, J. Zhu, *Adv. Funct. Mater*. **2021**, *31*, 2009869.

[8] R. C. Du, Z. C. Xu, C. Zhu, Y. W. Jiang, H. P. Yan, H. C. Wu, O. Vardoulis, Y. F. Cai, X. Y. Zhu, Z. N. Bao, Q. H. Zhang, X. D. Jia, *Adv. Funct. Mater.* **2020**, *30*, 1907139.

[9] Q. H. Zhang, S. M. Niu, L. Wang, J. Lopez, S. C. Chen, Y. F. Cai, R. C. Du, Y. X. Liu, J. C. Lai, L. Liu, C. H. Li, X. Z. Yan, C. G. Liu, J. B. H. Tok, X. D. Jia, Z. N. Bao, *Adv. Mater.* **2018**, *30,* 1801435.

[10] R. C. Du, Z. C. Xu, C. Zhu, Y. W. Jiang, H. P. Yan, H. C. Wu, O. Vardoulis, Y. F. Cai, X. Y. Zhu, Z. N. Bao, Q. H. Zhang, X. D. Jia, *Adv. Mater.* **2020**, *32*, 2000345.

[11] C. B. Cooper, S. Nikzad, H. P. Yan, Y. Ochiai, J. C. Lai, Z. A. Yu, G. Chen, J. Kang, Z. N. Bao, *Acs Central Sci.* **2021**, *7*, 1657-1667.

[12] J. H. Kang, D. Son, G. J. N. Wang, Y. X. Liu, J. Lopez, Y. Kim, J. Y. Oh, T. Katsumata, J. W. Mun, Y. Lee, L. H. Jin, J. B. H. Tok, Z. N. Bao, *Adv. Mater.* **2018**, *30,* 1706846.

[13] X. H. Wang, S. N. Zhan, Z. Y. Lu, J. Li, X. Yang, Y. N. Qiao, Y. F. Men, J. Q. Sun, *Adv. Mater.* **2020**, *32*, 2005759.

[14] Y. Song, Y. Liu, T. Qi, G. L. Li, *Angew. Chem. Int. Edit.* **2018**, *57,* 13838-13842.

[15] F. Y. Sun, J. H. Xu, T. Liu, F. F. Li, Y. Poo, Y. N. Zhang, R. H. Xiong, C. B. Huang, J. J. Fu, *Mater. Horiz.* **2021**, *8*, 3356-3367.

[16] O. Colombani, C. Barioz, L. Bouteiller, C. Chaneac, L. Fomperie, F. Lortie, H. Montes, *Macromolecules* **2005**, *38*, 1752-1759.

[17] F. Wang, Z. Yang, J. Li, C. Zhang, P. Sun, Acs Macro Letters **2021**, *10*, 510-517.

[18] H. J. Hu, R. Mo, X. Sheng, X. Zhang, *Polym. Chem.* **2020**, *11*, 2585-2594 (2020).

[19] Y. L. Sun, D. K. Sheng, H. H. Wu, X. X. Tian, H. P. Xie, B. R. Shi, X. D. Liu, Y. M. Yang, *Polymer* **2021**, *233*, 124208.

[20] W. Fan, Y. Jin, L. Shi, R. Zhou, W. Du, *J. Mater. Chem. A* **2020**, *8*, 6757-6767.

[21] W. X. Liu, C. Zhang, H. Zhang, N. Zhao, Z. X. Yu, J. Xu, *J. Am. Chem. Soc.* **2017**, *139*, 8678-8684.

[22] X. B. Zhu, W. J. Zhang, G. M. Lu, H. C. Zhao, *Acs Nano* **2022**, *16*, 16724-16735.

[23] M. N. Xie, D. K. Jia, J. Hu, J. Y. He, X. M. Li, R. J. Yang, *Polymers* **2021**, *13*, 152388.

[24] K. S. Kang, A. Phan, C. Olikagu, T. Lee, D. A. Loy, M. Kwon, H. J. Paik, S. J. Hong, J. Bang, W. O. Parker, *Angew. Chem. Int. Edit.* **2021**, *60*, 22900-22907.

[25] Q. Tang, R. Yang, J. He, *Ind. Eng. Chem. Res.* **2014**, *53*, 9714-9720.

[26] L. Xia, J. Liu, Z. Li, X. B. Wang, P. Wang, D. Wang, X. H. Hu, *J. Macromol. Sci. Part A-Pure Appl. Chem.* **2020**, *57*, 560-568.

[27] H. Chen, C. Deng, Z. Y. Zhao, L. Wan, A. H. Yang, Y. Z. Wang, *Chem. Eng. J.* **2020**, *400*, 125941.
